# Reconstitution of synaptic junctions orchestrated by teneurin-latrophilin complexes

**DOI:** 10.1126/science.adq3586

**Published:** 2025-01-16

**Authors:** Xuchen Zhang, Xudong Chen, Daniel Matúš, Thomas C. Südhof

**Affiliations:** Department of Molecular and Cellular Physiology, Howard Hughes Medical Institute, Stanford University, Stanford, CA 94305, USA

## Abstract

Synapses are organized by trans-synaptic adhesion molecules that coordinate assembly of pre- and postsynaptic specializations, which in turn are composed of scaffolding proteins forming liquid-liquid phase-separated condensates. Presynaptic teneurins mediate excitatory synapse organization by binding to postsynaptic latrophilins; however, the mechanism of action of teneurins, driven by extracellular domains evolutionarily derived from bacterial toxins, remains unclear. Here, we show that only the intracellular sequence, a dimerization sequence, and extracellular bacterial toxin-derived latrophilin-binding domains of Teneurin-3 are required for synapse organization, suggesting that teneurin-induced latrophilin clustering mediates synaptogenesis. Intracellular Teneurin-3 sequences capture liquid-liquid phase-separated presynaptic active zone scaffolds, enabling us to reconstitute an entire synaptic junction from purified proteins in which trans-synaptic teneurin-latrophilin complexes recruit phase-separated pre- and postsynaptic specializations.

Teneurins are enigmatic type II membrane proteins that are composed of an N-terminal cytoplasmic domain, a single transmembrane region (TMR), eight EGF-like repeats that form covalent cis-homodimers, an Ig-like domain, a β-propeller domain, a β-barrel domain, and a C-terminal toxin-like domain (Fig. 1A) (1–5). The four mammalian teneurin genes (referred to as Tenm1-4) can be considered an evolutionary puzzle because their C-terminal extracellular β-propeller, β-barrel, and toxin-like domains (that encompass >1,500 residues, accounting for >50% of the total protein) are derived by lateral gene transfer from two different bacterial toxins, TcB/TcC toxin subunits and group A colicins (Fig. 1A) (4–6). Teneurins perform multiple developmental roles, ranging from gastrulation and mesoderm induction (7, 8) to muscle development (9), neuronal migration (10), oligodendrocyte differentiation (11, 12), and synapse formation(13–18), but the mechanisms involved remain unclear.

Two hypotheses have been proposed. First, that teneurins act by a homophilic adhesion mechanism mediated by non-covalent trans-interactions of their β-propeller domains (19, 20). This hypothesis suggests that teneurin trans-homodimerization in combination with their covalent cis-homodimerization creates a trans-cellular signaling lattice. In particular, this mechanism is thought to determine the specificity of synaptic connections (3). Second, that teneurins act by a heterophilic adhesion mechanism mediated by tight binding to latrophilin adhesion-GPCRs that are key synaptic adhesion molecules (21, 22). Consistent with this hypothesis, only pre- but not postsynaptic deletions of Tenm3 and Tenm4 impaired hippocampal synaptic connections, whereas postsynaptic deletions of latrophilins suppressed hippocampal connections (23, 24).

The two mechanistic hypotheses about teneurins are supported by strong evidence but are mutually exclusive, rendering teneurin function as puzzling as their evolutionary history. The unusual architecture, unique evolution, and unclear functional mechanism of teneurins inspired us to investigate how teneurins promote formation of synaptic connections. We focused on Tenm3 using two complementary approaches, functional analyses of its individual domains and biochemical analyses of these domains in reconstituted systems. Our results reveal that Tenm3 acts in synapse formation as obligatory cis-homodimers that forms a trans-heterotetramers with latrophilins. We found that the essential N-terminal cytoplasmic Tenm3 sequence selectively recruits phase-separated presynaptic active zone scaffolds by direct binding to the active zone protein RIM1. These findings enabled us to reconstitute an entire synaptic junction complex composed of pre- and postsynaptic phase-separated scaffolds that are tethered to the Tenm3-Lphn3 assembly, suggesting a simple mechanism of synapse assembly whereby presynaptic Tenm3 cis-homodimers engage in a trans-complex with postsynaptic latrophilins to recruit pre- and postsynaptic phase-separated scaffolds to nascent synapses.

## A minimal teneurin protein drives synapse formation.

**Fig. 1.**
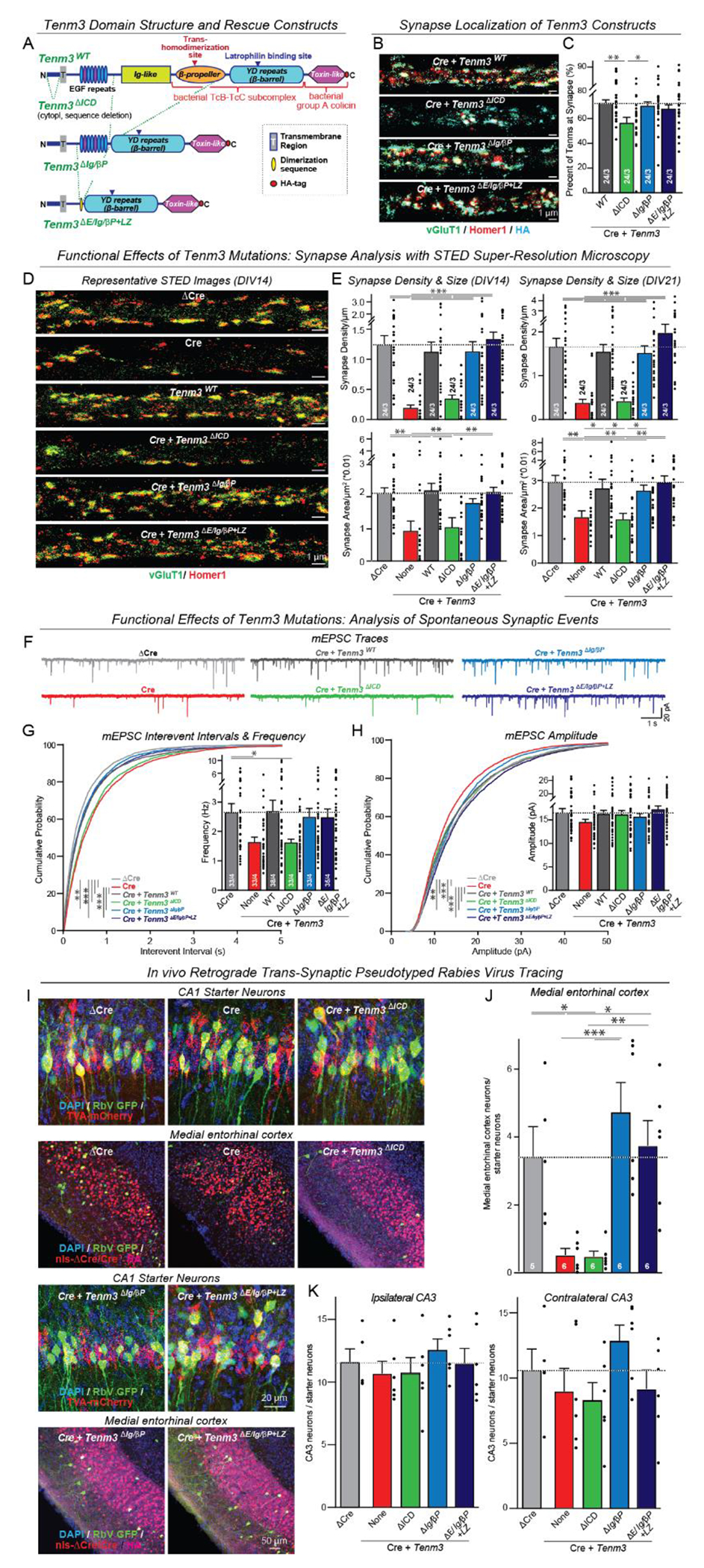
A minimal teneurin-3 (Tenm3) protein fully mediates synapse formation as long as latrophilin-binding is maintained (**A**) Domain structures of wild-type and mutant Tenm3 proteins. (**B** and **C**) STED super-resolution imaging of the synaptic localization of wild-type and mutant Tenm3 proteins expressed in cultured cortical neurons lacking Tenm3 and Tenm4 (B, representative images of dendrites stained for surface HA-tagged Tenm3 and the pre- and postsynaptic markers vGluT1 and Homer1; C, summary graphs of the synaptic Tenm3 levels, with synapses defined as puncta with coincident vGluT1- and Homer1-staining). (**D** and **E**) STED super-resolution imaging of rescue experiments of the synapse loss in Tenm3/4 double-deficient neurons. Cortical cultures from Tenm3/4 double cKO mice were infected with lentiviruses expressing the indicated Tenm3 proteins (ΔCre = mutant Cre) and analyzed after 14 (DIV14) or 21 days in culture (DIV21) (D, representative images; E, summary graphs of the synapse density and size at DIV14 and DIV21). For additional data including spine analyses, see figs. S1 to S9). (**F** to **H**) mEPSC measurements of the synapse loss rescue by minimal Tenm3 proteins in Tenm3/4 double-deficient cultured cortical neurons. mEPSCs were recorded at DIV14-16 in the presence of tetrodotoxin (1 μM) and picrotoxin (50 μM) (F, representative traces; G, cumulative plots of the interevent intervals and summary graphs of the mEPSC frequency; H, cumulative plots and summary graphs of the mEPSC amplitude). (**I** to **K**) *In vivo* trans-synaptic tracing experiments using pseudo-typed rabies viruses documenting that minimal Tenm3 proteins rescue the loss of entorhinal cortex→CA1 region synapses induced by the Tenm3/4 double deletion (I, representative images of postsynaptic starter neurons in the CA1 region (top) and of presynaptic ipsilateral entorhinal cortex input neurons (bottom); J and K, quantification of synaptic input neurons in the ipsilateral entorhinal cortex (J), ipsilateral CA3 region (K, left; negative control), and contralateral CA3 region (K, right; additional negative control)). Numerical data are means ± SEM (numbers of cells, experiments, and mice are indicated in bars). ***P < 0.001, **P < 0.01, *P < 0.05 [One-way ANOVA with post hoc Tukey/Dunnett tests; G and H, Kolmogorov–Smirnov t test for cumulative distributions]; a.u., arbitrary units. For additional data, see figs. S1 to S10.

The structure of teneurins in which more than half of their extracellular domains are derived from bacterial toxins (Fig. 1A) prompts the question which of these domains are functionally important. Therefore, we performed rescue experiments in cultured neurons from Tenm3 and Tenm4 double-conditional KO mice (Tenm3/4 cKO mice) (25). Conditional deletion of Tenm3 and Tenm4 caused a partial loss of dendritic spines and excitatory synapses in cultured neurons without changing their axonal or dendritic arborizations (figs. S1 and S2), thus making this culture system suitable for mechanistic analyses of teneurins in synapse formation.

We generated 17 mutant Tenm3 constructs containing a C-terminal HA-tag (fig. S3). When expressed in neurons, all Tenm3 constructs were robustly expressed on the neuronal surface and targeted to synapses, albeit with varying efficiency (Fig. 1B and C, and fig. S4). Immunoblotting analyses revealed that Tenm3 was constitutively cleaved in neurons, causing secretion of a >250 kDa fragment (fig. S5). Cleavage was blocked when the Ig-like and β-propeller domains were deleted, suggesting a cleavage site between the EGF-like and Ig-like domains. We did not observe C-terminal cleavage producing a ‘Teneurin C-Terminal Associated Peptide’ (TCAP) (26) (fig. S5).

We next investigated which Tenm3 domains were minimally required to reverse the synapse loss caused by the Tenm3/4 deletion. Synapse density measurements, performed by immunocytochemistry and confocal and STED super-resolution microscopy, revealed that deletion of the two major central domains of Tenm3 (the Ig-like and β-propeller domains [residues 784-1517]) did not impair the synaptic function of Tenm3, whereas deletion of the N-terminal intracellular domain (ICD) blocked Tenm3 function (Fig. 1D and E, and figs. S6A, S7A and B). We commonly monitor synapse densities after 14 days in culture (DIV14), which may produce an overestimate of a phenotype if it is transient. Therefore, we also measured the synaptic activity of key constructs at DIV21. We detected the same phenotypes (Fig. 1E and fig. S6B), thus confirming that the phenotype and its rescues were not developmentally limited.

Deletion of the EGF-like domains of teneurins that form disulfide-bonded homodimers (1, 27) also abolished rescue similar to the Tenm3 ICD deletion, as did point mutations in the cysteines of the EGF-like domains that mediate their covalent dimerization (fig. S7C to I). To test whether the only role of the EGF-like domains is to mediate Tenm3 cis-dimerization, we replaced the entire 8 EGF-like domains spanning 259 residues [residues 514-773] with a short 41-residue exogenous non-covalent dimerization sequence (Fig. 1A and fig. S3A). The exogenous cis-dimerization sequence fully restored the rescue of synapse loss after deletion of the EGF-like domains (Fig. 1D and E, and figs. S6A and S7C to I), suggesting that the only obligatory function of the EGF-like domains is dimerization. The minimal Tenm3 construct that is functionally active in synapse formation (the Tenm3^ΔE/Ig/βP+LZ^ construct that lacks the EGF-like repeat, Ig-like and β-propeller domains but contains an artificial dimerization sequence) operates by binding to latrophilins, since a point mutation in the latrophilin-binding sequence (Tenm3^ΔE/Ig/βP+LZ^*) abolished its function in synapse formation (fig. S7E to I). Moreover, even small deletions from the N-terminal ICD of Tenm3 impaired its synaptic function (fig. S7L and M), as did small deletions from the C-terminal toxin-like domain of Tenm3 (fig. S8A to C). Finally, overexpression of Tenm3 constructs in neurons with normal expression of endogenous Tenm3 and Tenm4 had no effect on synapse numbers (fig. S9), indicating that different from neuroligins (28), overexpression of Tenm3 by itself does not cause a dominant effect.

To independently confirm the immunocytochemical synapse density measurements, we performed Ca^2+^-imaging experiments (fig. S10) and recordings of spontaneous miniature excitatory postsynaptic currents (mEPSCs) (Fig. 1F to H, and fig. S6C). Ca^2+^-imaging showed that the Tenm3/4 deletion decreased the frequency of spontaneous network activity in cultured neurons. This phenotype was fully rescued by the minimal Tenm3 construct lacking the EGF-like, Ig-like, and β-propeller domains and containing a dimerization sequence, but not by Tenm3 with a deletion of its intracellular domain (ICD) (fig. S10). Similarly, mEPSC measurements revealed that the Tenm3/4 deletion caused a large decrease (~50%) in mEPSC frequency that was rescued by the minimal Tenm3 construct but not by the Tenm3 mutant lacking the ICD (Fig. 1F to H, and fig. S6C). We observed no major changes in the mEPSC amplitude or intrinsic electrical properties of the neurons (Fig. 1H; fig. S6C), consistent with a selective decrease in synapse numbers. Thus, more than 1,000 residues of Tenm3 are dispensable for synapse formation as long as Tenm3 cis-dimerization, latrophilin binding, and its ICD are maintained. These findings indicate that Tenm3 acts in synapse formation by dimerizing latrophilins, whereas the trans-homodimerization of teneurins observed in atomic structures that is mediated by their β-propeller domain (29, 30) has no functional role in synapse formation.

## In vivo validation.

Culture experiments investigating synapse formation can be misleading given the promiscuity of synaptic connections between cultured neurons. We thus asked whether the findings in cultured neurons also apply to *in vivo* conditions. We previously showed using a modified retrograde trans-synaptic pseudo-typed rabies virus tracing method (31) that deletion of presynaptic Tenm3/4 from entorhinal cortex neurons suppresses their synaptic connections with CA1-region target neurons (25). Here, we confirmed this observation, with a >90% suppression of synaptic connections (Fig. 1I to K, and fig. S11). This phenotype was completely rescued by presynaptic expression of the minimal Tenm3 construct which lacks the EGF-like, Ig-like, and β-propeller domains but contains an exogenous dimerization sequence (Fig. 1I to K, and fig. S11). The ICD deletion of Tenm3 again abolished the rescue. Thus, Tenm3 organizes synapses *in vivo* by connecting the action of its presynaptic ICD to the postsynaptic clustering of latrophilins that is mediated by its bacterially derived toxin-like domains.

## The functionally essential Tenm3 intracellular domain captures phase-separated active-zone scaffolds.

**Fig. 2.**
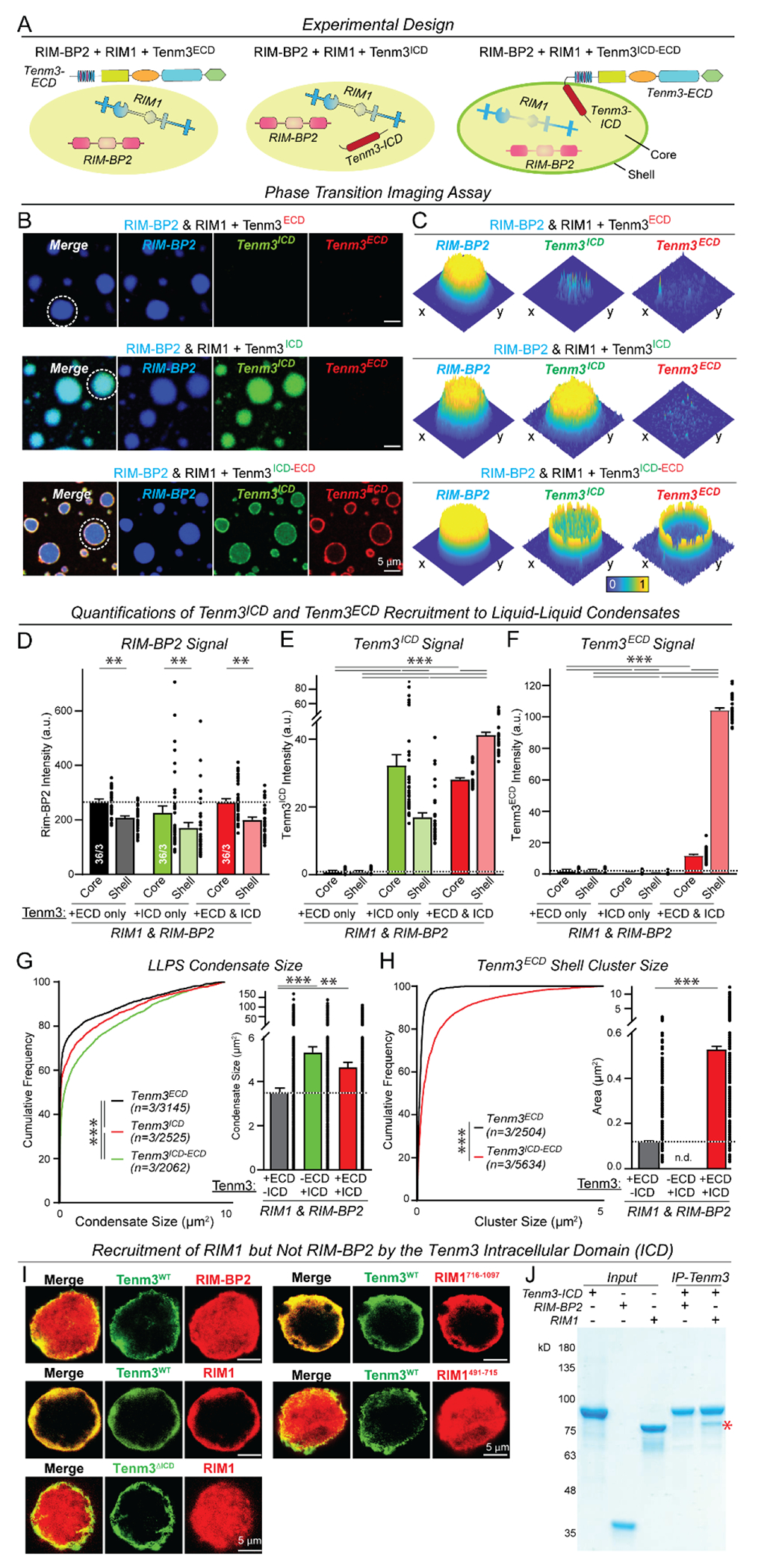
The intracellular domain (ICD) of Tenm3 incorporates into active zone liquid-liquid phase-separated (LLPS) condensates and recruits the Tenm3 extracellular domains (ECDs) to the surface shell of active zone LLPS condensates (**A**) Experimental design of LLPS experiments. RIM1 and RIM-BP2 forming presynaptic active zone LLPS condensate via phase separation (40) were analyzed with addition of the Tenm3 ECD (Tenm3^ECD^), ICD (Tenm3^ICD^), or full-length Tenm3 (Tenm3^ECD-ICD^; fig. S12). (**B**) Representative images illustrating the recruitment of Tenm3^ICD^ and Tenm3^ECD-ICD^ but not of Tenm3^ECD^ (all 1 μM) to RIM1 (10 μM) and RIM-BP2 (10 μM) active zone LLPS condensate (RIM-BP2, Tenm3^ECD^, and Tenm3^ICD^ were labeled by iFluor-405, iFluor-546 and iFluor-488, respectively, while RIM1 was unlabeled). (**C**) Representative heatmaps of the enrichment of RIM-BP2, Tenm3^ICD^, and Tenm3^ECD^ signals in active zone LLPS condensates (examples indicated by dashed circles in B). (**D** to **F**) Quantification of RIM-BP2, Tenm3^ICD^, and Tenm3^ECD^ signals across phase-separated active zone LLPS condensates illustrating that addition of Tenm3 has no effect on the distribution of RIM-BP2 in the condensates (D), that Tenm3^ICD^ localizes to the condensate core in the absence of the ECDs but to the condensate shell when coupled to ECDs in Tenm3^ECD-ICD^ proteins (E), and that the Tenm3 ECDs are only present on the condensate surface when coupled to the Tenm3 ICD in Tenm3^ECD-ICD^ protein (F). (**G**) Quantification of the size of LLPS condensates formed by active zone proteins RIM1 and RIM-BP2 in the presence of Tenm3^ECD^, Tenm3^ICD^, and Tenm3^ICD-ECD^. (**H**) Quantification of Tenm3^ECD^ and Tenm3^ICD-ECD^ shell cluster sizes on the surface of RIM1/RIM-BP2 active zone LLPS condensates. (**I**) Imaging of transfected HEK293T cells co-expressing Flag-tagged RIM1 or RIM-BP2 constructs with HA-tagged Tenm3^WT^ or Tenm3^ΔICD^ (red, Flag-epitope; green, HA-epitope). (**J**) Co-immunoprecipitation experiments of purified Tenm3^ICD^, RIM1, and RIM-BP2 confirm that the Tenm3^ICD^ directly binds to RIM1 but not RIM-BP (Coomassie-stained SDS-gel (input (In), 5% of total; asterisk, co-immunoprecipitated RIM1). Numerical data are means ± SEM (numbers of condensates and experiments are indicated in bars). ***P < 0.001, **P < 0.01, *P < 0.05 [D, E, and F: Two-way ANOVA with post hoc Tukey tests; G and H left: Kolmogorov–Smirnov t test; H right: One-way ANOVA with post hoc Tukey tests]; a.u., arbitrary units.

We recently reported that during synapse formation, the latrophilin-3 (Lphn3) cytoplasmic sequence recruits postsynaptic scaffold proteins as liquid-liquid phase separated (LLPS) condensates (32). Could Tenm3 correspondingly recruit presynaptic active-zone scaffold proteins present as LLPS condensates? Purified recombinant RIM1 and RIM-BP2 scaffold proteins that are central components of active zone complexes (33) avidly formed LLPS condensates (Fig. 2A to C) as described (34, 35). When we added the purified recombinant Tenm3 ICD to the active zone LLPS condensates, the ICD fully integrated into and filled out the entire LLPS condensates (Fig. 2A to F, and figs. S12 and S13). When we added full-length Tenm3 protein (produced by protein ligation of the Tenm3 extracellular domains (ECDs) to the Tenm3 ICD (fig. S12E to G)), the Tenm3 ICD still integrated into the phase-separated active zone scaffolds but, together with the Tenm3 ECDs, formed a shell on the surface of the LLPS condensate core (Fig. 2A to F, and fig. S13). The shell formed by full-length Tenm3 on active-zone LLPS condensates did not cause major changes in LLPS condensate size (Fig. 2G) and segregated into clusters (Fig. 2H) (25). All phase-separation processes with recruitment of the Tenm3 ICD were independent of the pH (fig. S14).

Since we used a full-length Tenm3 protein lacking a hydrophobic TMR in these experiments, the question arises whether the Tenm3 ICD also mediates recruitment of active-zone LLPS condensates when attached to a lipid bilayer. To address this question, we tethered the Tenm3 ICD to the surface of giant unilamellar vesicles. The Tenm3 ICD also robustly recruited the active-zone LLPS condensates to the lipid bilayer surface (fig. S15 and movies S1 and S2), suggesting that the Tenm3 ICD efficiently tethers LLPS condensates to the surface of a hydrophobic lipid bilayer.

We next asked whether the Tenm3 ICD binds directly to RIM1 and/or RIM-BP2 in the active-zone LLPS condensates. Using membrane-recruitment assays in transfected HEK293 cells, we found that RIM1 but not RIM-BP2 directly binds to the ICDs of all four teneurins (Fig. 2I and fig. S16A). Only the central sequences of RIM1 (residues 491-715) bind to teneurin ICDs (fig S16B). Selective binding of the Tenm3 ICD to RIM1 but not RIM-BP2 was confirmed using purified recombinant proteins as an independent approach (Fig. 2J).

**Fig. 3.**
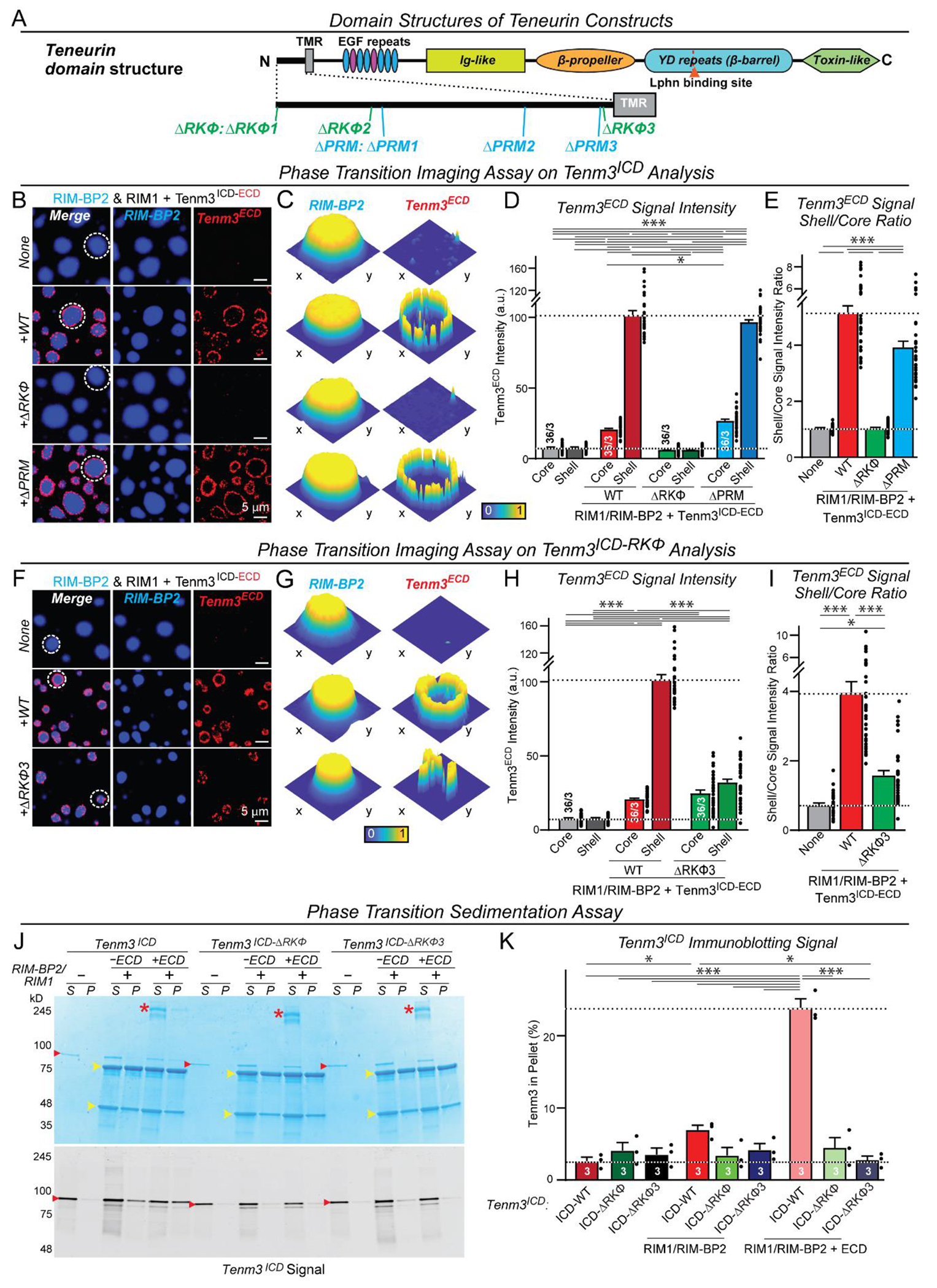
Recruitment of the Tenm3 ICD into active zone LLPS condensates is sequence-specific (**A**) Design of mutations in the Tenm3 ICD (see figs. S11 and S16 for sequence information). (**B** and **C**) Representative images (left) and heatmaps (right, corresponding to the circled LLPS active zone condensates on the left) demonstrating the effect of the ΔRKΦ and ΔPRM mutations on the recruitment of the Tenm3^ECD-ICD^ to the shell of active zone LLPS condensates. Condensates composed of RIM1 and RIM-BP2 (both 20 μM, RIM-BP2 is labeled with iFluor-405) were incubated without or with wild-type or mutant Tenm3^ICD-ECD^ (1 μM; ECD is labeled with iFluor-546). (**D** and **E**) Quantification of Tenm3^ICD-ECD^ recruitment to the shell of presynaptic active zone condensates as a function of ICD mutations (left, absolute signal intensity; right, ratio of shell to core signal with ‘control’ signals constituting background). (**F** and **G**) Representative images (left) and heatmaps (right, corresponding to circled active zone LLPS condensates on the left) demonstrating that the effect of the small ΔRKΦ3 deletion on the Tenm3^ECD-ICD^ protein recruitment to active zone LLPS condensates. Conditions were the same as in B and C. (**H** and **I**) Quantification of Tenm3^ICD-ECD^ recruitment to the shell of presynaptic active zone condensates as a function of the RKΦ3 mutation (left, absolute signal intensity; right, ratio of shell to core signal with ‘control’ signals constituting background). (**J**) Sedimentation assay of LLPS active zone condensates documenting that the RKΦ3 mutation blocks Tenm3 recruitment to condensates (top, Coomassie-stained gel; bottom, immunoblot [yellow arrowheads, RIM1 and RIM-BP2; red arrowheads, Tenm3^ICD^; asterisks, Tenm3^ICD-ECD^]). Active zone LLPS condensates composed of RIM1 and RIM-BP2 (both 20 μM) were incubated without or with wild-type or mutant Tenm3^ICD^ or Tenm3^ICD-ECD^ (1 μM) and pelleted by centrifugation (P, pellets; S, supernatants). (**K**) Quantification of the recruitment of Tenm3^ICD^ and Tenm3^ICD-ECD^ to active zone LLPS condensates as measured by the sedimentation assay. Summary graph depicts the percentage of Tenm3 in the pellet as measured by quantitative immunoblotting. Numerical data are means ± SEM (numbers of condensates and experiments are indicated in bars). ***P < 0.001, **P < 0.01, *P < 0.05 [D, H, and K: Two-way ANOVA with post hoc Tukey tests; E and I: One-way ANOVA with post hoc Tukey tests]; a.u., arbitrary units.

Is the recruitment of phase-separated active-zone scaffolds and the binding of RIM1 by the Tenm3 ICD sequence-specific? Comparative analyses of teneurin sequences revealed three conserved proline-rich motifs (PRMs) and three regions of high arginine, lysine, and aromatic amino acid content (RKΦs) that are known to promote phase separation (36, 37) (Fig. 3A and fig. S17A, B, K and L). Deletion of the proline-rich ‘PRM’ sequences had no effect on the integration of the Tenm3 ICD into active-zone LLPS condensates, suggesting these sequences are dispensable (Fig. 3B to D and fig. S17C to F). Deletion of the arginine-lysine-rich ‘RKΦ’ sequences, however, blocked integration of the Tenm3 ICD into active-zone LLPS condensates (Fig. 3B to E and fig. S17C to F). Moreover, deletion of just one of the three RKΦ regions (RKΦ3) severely impaired active-zone LLPS condensate integration (Fig. 3F to I and fig. S17G to J). These conclusions were based on two independent methods: imaging (Fig. 3B to I) and centrifugation (Fig. 3J and K). Moreover, the same mutations in the Tenm3 ICD that abolished its recruitment into active-zone LLPS condensates also impaired its binding to RIM1, whereas the mutations deleting the proline-rich regions had no effects (fig. S18). Consistent with the direct binding of the Tenm3 ICD to RIM1, RIM1-only LLPS condensates (40) also recruited full-length Tenm3 with a wild-type ICD but not Tenm3 with an ‘RKΦ’ mutant ICD (fig. S19). Thus, recruitment of active-zone LLPS scaffolds to Tenm3 via its binding to RIM1 is sequence-specific.

## Recruitment of active-zone LLPS condensates by the Tenm3 ICD is required for synaptic function.

Compelling evidence supports the physiological importance of pre- and postsynaptic LLPS condensates (34, 35, 38–42), but their functional relevance remains incompletely understood. Thus, we asked whether rescue of the impaired synaptic connectivity in Tenm3/4-deficient neurons requires the ability of Tenm3 to recruit active-zone LLPS condensates.

**Fig. 4.**
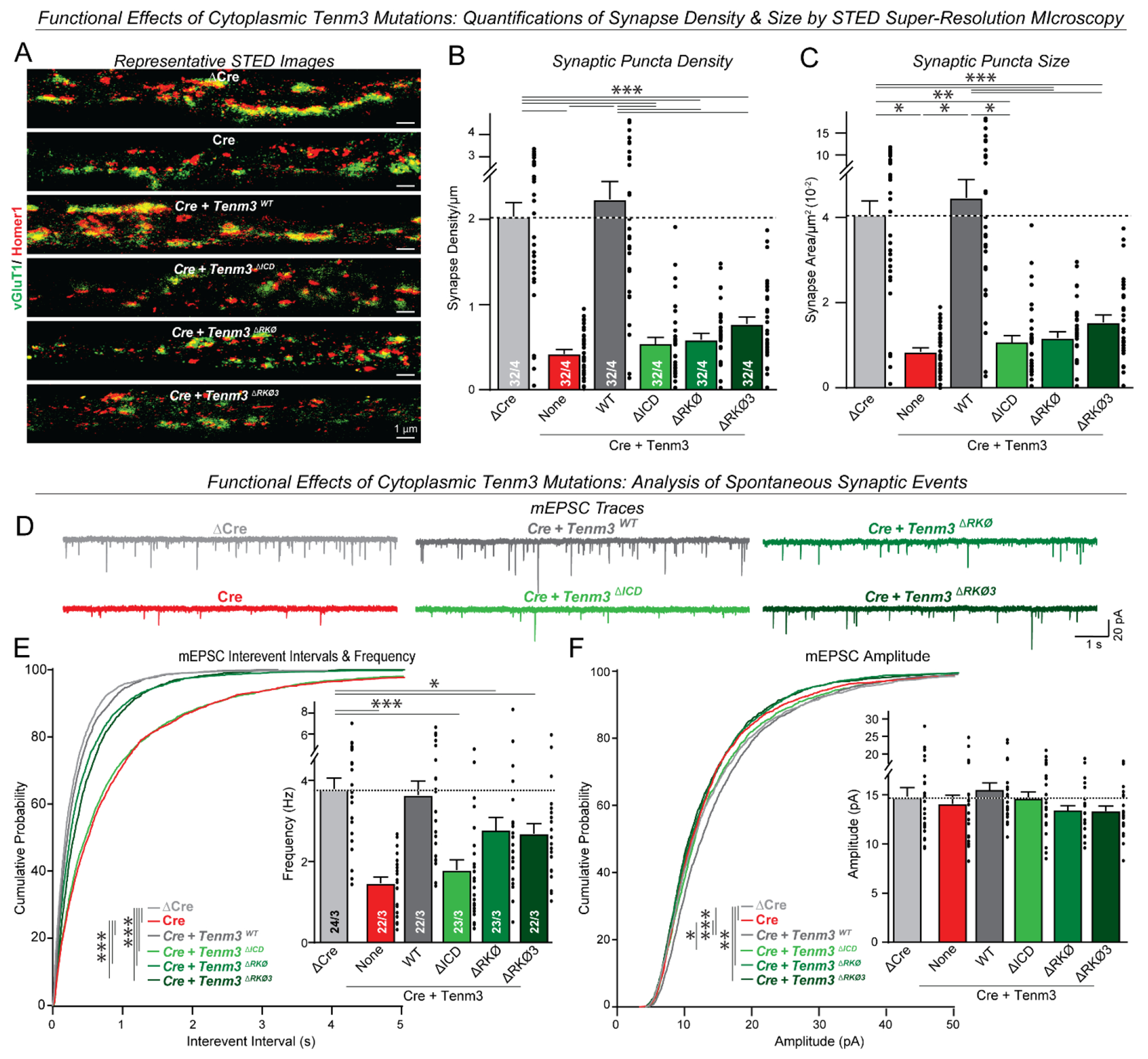
Tenm3 ICD mutations that disrupt recruitment of the ICD to active zone LLPS condensates abolish Tenm3 function in synapse formation. (**A** to **C**) STED super-resolution imaging demonstrating that mutations of Tenm3 that delete its intracellular domain (Tenm3^ΔICD^), its RKΦ regions (Tenm3^ΔRKΦ^), or its RKΦ3 sequence (Tenm3^ΔRKΦ3^) abolish the ability of Tenm3 to rescue decreased excitatory synapse numbers and sizes in Tenm3/4-deficient neurons (A, representative images; B and C, summary graphs of the synapse density and size, respectively). (**D** to **F**) The same mutations as analyzed in A-C also abolish the ability of Tenm3 to rescue the decreased mEPSC frequency of Tenm3/4-deficient cultured neurons at DIV14-16 (D, representative traces; E, summary graphs of the mEPSC frequency and cumulative plots of the interevent intervals; F, summary graphs and cumulative plots of the mEPSC amplitude). Numerical data are means ± SEM (numbers of cells and experiments are indicated in bars). ***P < 0.001, **P < 0.01, *P < 0.05 [One-way ANOVA with post hoc Tukey/Dunnett tests; E and F, Kolmogorov–Smirnov t test for cumulative distributions].

Using two independent assays, immunocytochemical measurements of synapse density aided by STED super-resolution microscopy and recordings of mEPSCs, we found that the RKΦ and the RKΦ3 mutations that disrupt incorporation of the Tenm3 ICD into phase-separated active-zone LLPS condensates also block rescue of the synapse loss induced by the Tenm3/4 deletion (Fig. 4 and fig. S20). As in Fig. 1, only the mEPSC frequency but not the mEPSC amplitude was suppressed by the Tenm3/4 deletions without changes in the intrinsic electrical properties, as expected for a synapse loss phenotype (Fig. 4 and fig. S20B). The morphologically measured synapse loss appeared to be more severe than the decrease in mEPSC frequency in these experiments, possibly because the remaining synapses are more active. Viewed together, these findings support the hypothesis that recruitment of active-zone LLPS condensates is critical for synapse formation.

## Reconstitution of a synaptic junction from purified proteins.

**Fig. 5.**
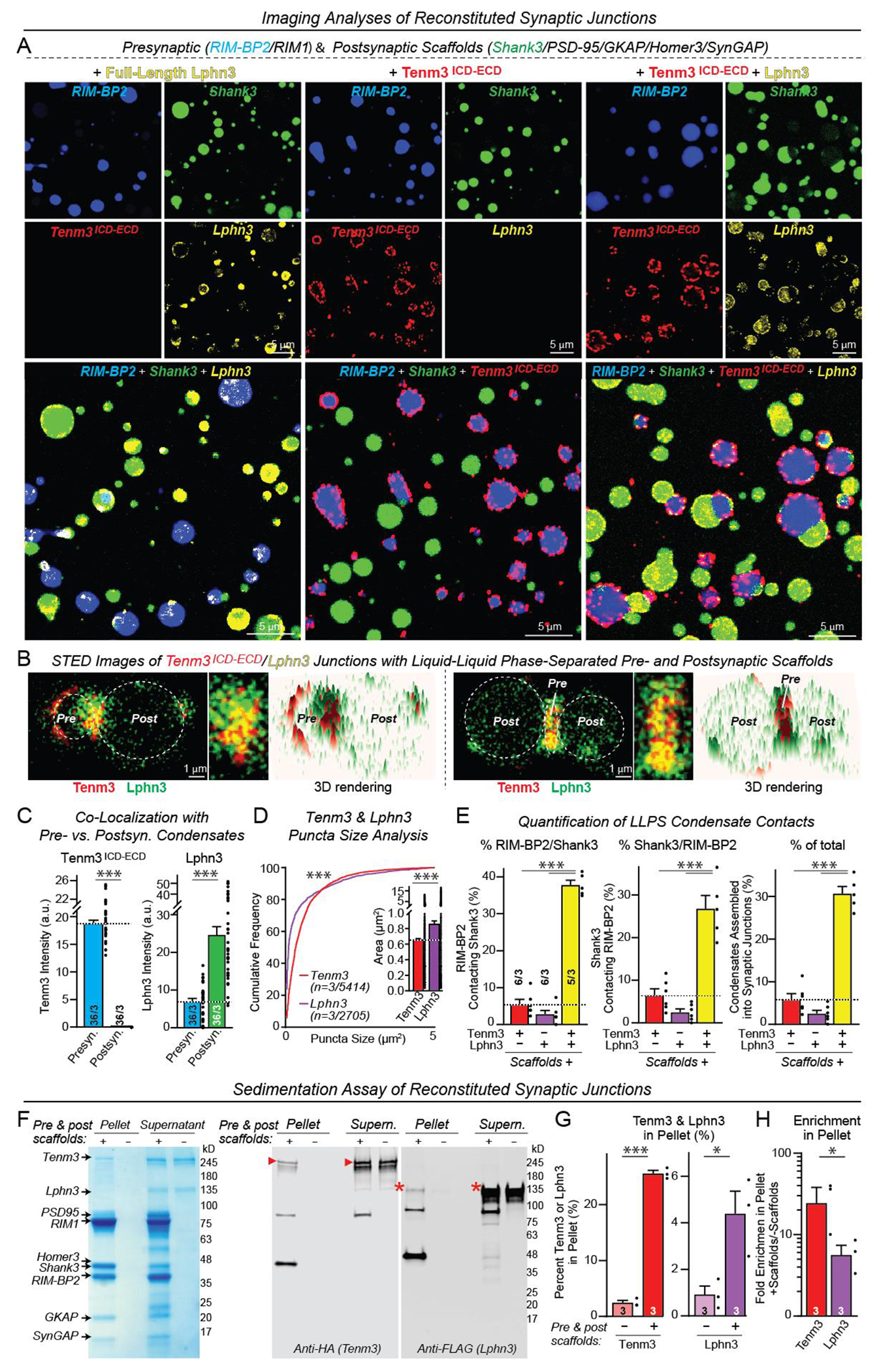
Reconstitution of synaptic junctions from purified proteins enabled by the Lphn3-Tenm3 complex that selectively recruits presynaptic active zone and postsynaptic LLPS condensates (**A**) Representative images of phase-separated active zone and postsynaptic density LLPS condensates with addition of full-length Tenm3^ICD-ECD^ and/or Lphn3 (both at 0.5 μM). Active zone condensates were formed with RIM1 and RIM-BP2 (both 20 μM) and PSD condensates with PSD-95, Homer3, truncated GKAP with a DLS sequence at the GK binding region (a phospho-mimicking mutation to enhance PSD-95 binding (38), SynGAP, and Shank3 (all 2.5 μM) (32, 42). (**B**) High-magnification images of representative reconstituted synaptic junctions that are formed by phase-separated active zone and postsynaptic density LLPS condensates containing Tenm3^ICD-ECD^ (red) and Lphn3 (green), respectively (left, STED images; right, 3D-rendering heatmaps of Tenm3^ICD-ECD^ and Lphn3 levels). (**C**) Quantification of the co-localization of Tenm3^ICD-ECD^ and Lphn3 with active zone or postsynaptic LLPS condensates documenting that Tenm3^ECD-ICD^ and Lphn3 are selectively recruited to active zone or postsynaptic density condensates, respectively, even when these condensates are mixed. (**D**) Cumulative frequency plots of the sizes of the Tenm3^ICD-ECD^ and Lphn3 puncta on the LLPS condensate surfaces (inset, summary graph of mean sizes). (**E**) Quantifications of active zone and postsynaptic LLPS condensate contacts. Summary graphs show the percentages of active zone condensates contacting PSD condensates (left), of PSD condensates contacting active zone condensates (middle), and of total condensates forming contacts (right). (**F**) Sedimentation assays confirming reconstitution of synaptic junctions. Tenm3^ICD-ECD^ and Lphn3 were incubated in the absence and presence of mixtures of pre- and postsynaptic LLPS condensates that were formed as in panel A and centrifuged. The supernatants and pellets were analyzed by SDS-PAGE and Coomassie staining (left) or immunoblotting (right). (**G**) Quantification of the pellet enrichment of Tenm3^ICD-ECD^ and Lphn3 in the absence and presence of pre- and postsynaptic LLPS condensates. (**H**) Quantification of the fold enrichment of Tenm3^ICD-ECD^ and Lphn3 in the pellet in the absence and presence of pre- and postsynaptic scaffold protein LLPS condensates. Numerical data are means ± SEM (numbers of condensates and experiments are indicated in bars). ***P < 0.001, **P < 0.01, *P < 0.05 [C, G, H, and D inset: two-tailed t test; D: Kolmogorov–Smirnov t test; E: One-way ANOVA with post hoc Tukey tests]. a.u., arbitrary units.

Is the recruitment of phase-separated active-zone scaffolds by the Tenm3 ICD specific for active-zone LLPS condensates or does a similar interaction occur with all types of LLPS condensates? Moreover, might it be possible to reconstitute an entire trans-synaptic junction from purified proteins since Tenm3 and Lphn3 both recruit phase-separated protein scaffolds that are specific for pre- or postsynaptic specializations? To address these key questions, we generated presynaptic active-zone and postsynaptic scaffold LLPS condensates. We then mixed these condensates with each other and with purified full-length Lphn3 and Tenm3 that form a complex to test whether they form reconstituted junctions (Fig. 5).

Imaging analyses revealed that the pre- and postsynaptic scaffolds assembled into separate LLPS condensates even when combined, demonstrating a high degree of specificity in liquid-liquid phase separation (Fig. 5A and fig. S21). Tenm3 and Lphn3 exclusively recruited pre- or postsynaptic LLPS condensates, respectively, uncovering a selectivity in their interactions with phase-separated scaffolds (Fig. 5A to C, and fig. S22). The Tenm3-Lphn3 complex was localized to the contact points between the phase-separated condensates where they formed a junction (Fig. 5A to E, and figs. S21 and S23). In addition, uncomplexed Tenm3 and Lphn3 were present in the shell of their cognate pre- or postsynaptic LLPS condensates, respectively, outside of junctional complexes. The overall dimensions of the reconstituted synaptic junctions were similar to those of neuronal synapses with a diameter of ~1 μm (fig. S21L). Sedimentation assays of LLPS condensates confirmed the recruitment of Tenm3-Lphn3 complexes to pre- and postsynaptic scaffold protein condensates (Fig. 5F to H). Thus, we have reconstituted a synaptic junction complex that includes assembly of specific phase-separated pre- and postsynaptic scaffolds recruited by interacting cell-adhesion molecules.

## Summary.

Our results reveal a simple and economical mechanism of teneurin function in synapse formation that consists of the recruitment of phase-separated pre- and postsynaptic scaffolds by trans-synaptic Tenm3 and Lphn3 complexes. We uncovered previously unappreciated features of this mechanism: first, we found that nearly half of the extracellular Tenm3 domains are functionally dispensable for synapse formation, although it is possible that the dispensable domains perform other functions not analyzed here. The non-essential domains include the Tenm3 β-propeller domain that mediates their homophilic interactions, which are thus not physiologically relevant in synapse formation, whereas latrophilin binding is. Second, we show that Tenm3 function in synapse formation requires homophilic cis-dimerization of Tenm3 that is normally mediated by their EGF-like domains via a covalent disulfide bond but can be replaced by a non-covalent leucine-zipper dimerization sequence. Thus, teneurins need to cluster latrophilins for synapse formation. Third, the long cytoplasmic Tenm3 sequence (its ICD) fully inserts into phase-separated presynaptic active-zone LLPS condensates via direct binding to RIM1, with a single mutation in the Tenm3 ICD disrupting the recruitment of phase-separated active zone scaffolds and RIM1 binding. The recruitment of phase-separated active zone condensates by Tenm3 is functionally essential for synapse formation. Fourth, in the reconstitution of the trans-synaptic complex containing pre- and postsynaptic phase-separated scaffolds, the scaffolds spontaneously assembled into distinct phases that do not mix and are separately selectively recruited by their cognizant adhesion molecules. Thus, phase separation of pre- and postsynaptic scaffolds exhibits a high degree of specificity that could account for the amazing selectivity of the protein compositions of synaptic junctions. Together, these findings suggest that the trans-synaptic teneurin-latrophilin complex nucleates synapse assembly by recruiting pre- and postsynaptic LLPS scaffolds to nascent synaptic junctions.

Our study is limited in that we focused on Tenm3, we only examined a single function of Tenm3 (synapse formation) even though teneurins have multiple other biological roles (8–15), and we restricted our studies to hippocampal and entorhinal cortex synapses. It is possible that other teneurins function by different mechanisms despite their similarity and that teneurins act by distinct mechanisms in diverse functions. Moreover, our biochemical reconstitution of synaptic junctions only examined a few proteins and did not include many other important proteins (33, 43–45). Finally, we did not study other synaptic adhesion-signaling mechanisms, such as those mediated by neurexins, LAR-type PTPRs, EphB’s, or FLRTs (46–55). Despite these limitations, our study provides valuable insights on how synapses are built that describe a direct mechanism by which active zone scaffolds are recruited to synapses and report the first reconstitution of a synaptic junction complex from purified recombinant proteins.

## Supplementary Material

moive S1

moive S2

MDAR Reproducibility Checklist

SOMs

## Data Availability

All raw data and code supporting the findings of this study have been deposited in the Stanford Data Repository (https://purl.stanford.edu/xx501gx1493).
