# Supplementary material for: Reconstitution of synaptic junctions orchestrated by teneurin-latrophilin complexes
Source: Science. Author manuscript; Available in PMC 2025 Feb 10. (PMC11808628; doi:10.1126/science.adq3586)
Supplement: SOMs [file NIHMS2049802-supplement-SOMs.pdf]

Science
AAAS

Supplementary Materials for

Reconstitution of synaptic junctions orchestrated by teneurin-latrophilin complexes

Xuchen Zhang†*, Xudong Chen†, Daniel Matúš, and Thomas C. Südhof*

*Corresponding authors. E-mail addresses: zxc1@stanford.edu; tcs1@stanford.edu

The PDF file includes:

Materials and Methods
Figs. S1 to S23
References

Other Supplementary Materials for this manuscript include the following:

Legend for Supplementary Movies S1 and S2

Materials and Methods

Mouse Breeding, Genotyping, and Husbandry. *Tenm3/4* DcKO mice were used as described (*25*). Mice were weaned at 20-22 days of age and group-housed (less than 5 mice per cage) on a 12 h light-dark cycle with food and water ad libidum in the Stanford Veterinary Service Center, both male and female animals were used for all experiments. Stanford Animal Housing Facility: All procedures conformed to National Institutes of Health Guidelines for the Care and Use of Laboratory Mice and were approved by the Stanford University Administrative Panel on Laboratory Animal Care. Hippocampal and entorhinal cortex from male and female newborn (P0) mice were combined to generate primary mixed neuron-glia cultures; both male and female mice were randomly used for monosynaptic retrograde rabies tracing experiments.

Plasmids and Viruses. For monosynaptic retrograde rabies tracing experiments, the single AAVs helper vector AAV-TCB-mCherry-P2A-RG was constructed and used as before (*25*). The glycoprotein-deleted rabies virus (EnvA) was produced by the Janelia Farm Viral Core Facility. Lentiviruses expressing NLS-EGFP/tdTomato-ΔCre or NLS-EGFP/tdTomato-Cre driven by the human Synapsin promoter (*56*) and used for all neuronal culture experiments. Tenm3 rescue plasmids were based on mouse full-length cDNA and contained an HA tag at the C-terminus with a GG linker. The domain structures of the various encoded proteins are shown in fig. S3. The following viruses were generated and used in various experiments:

AAV-Syn-NLS-tdTomato-ΔCre

AAV-Syn-NLS-tdTomato-Cre

AAV-Syn-TCB-mCherry-P2A-RG

RbV-CVS-N2c-deltaG-GFP (EnvA)

Lenti-hSyn-NLS-EGFP-ΔCre

Lenti-hSyn-NLS-EGFP-Cre

Lenti-hSyn-NLS-tdTomato-ΔCre

Lenti-hSyn-NLS-tdTomato-Cre

Lenti-hSyn-GCaMP6m

Lenti-EF1α-Tenm3-*WT*

Lenti-EF1α-Tenm3-*ΔICD*

Lenti-EF1α-Tenm3-*ΔIg/βP*

Lenti-EF1α-Tenm3-*ΔIg/βP**

Lenti-EF1α-Tenm3-*ΔE/Ig/βP*

Lenti-EF1α-Tenm3-*ΔE/Ig/βP+LZ*

Lenti-EF1α-Tenm3-*ΔE/Ig/βP+LZ**

Lenti-EF1α-Tenm3-*ΔIg/βP CS mt1*

Lenti-EF1α-Tenm3-*ΔIg/βP CS mt2*

Lenti-EF1α-Tenm3-*ΔIg/βP CS mt3*

Lenti-EF1α-Tenm3-*ΔE/Ig/βP+LZ-TxS mt1*

Lenti-EF1α-Tenm3-*ΔE/Ig/βP+LZ-TxS mt2*

Lenti-EF1α-Tenm3-*ΔE/Ig/βP+LZ-TxS mt3*

Lenti-EF1α-Tenm3-*ΔE/Ig/βP+LZ-TxS mt4*

Lenti-EF1α-Tenm3-ICD-*ΔRKΦ*

Lenti-EF1α-Tenm3-ICD-*ΔPRM*

Lenti-EF1α-Tenm3-ICD-*ΔRKΦ3*

See fig. S3 and references (*10, 57*) for a description of the encoded protein constructs.

Lentivirus and AAV Preparations. Lentiviruses and AAVs were prepared as described (*25, 58*). For productions of AAVs, briefly, AAV-DJ expressing vectors were co-transfected with two helper plasmids (pHelper and pRC-DJ) into HEK293T cells using calcium phosphate, at 200 µg of each plasmid per 150 cm^2 culture area. Transfected cells were collected, lysed, and loaded into an iodixanol gradient for ultracentrifugation (400,000×g for 3 h). The 40% iodixanol fraction with the virus was further washed and concentrated with a 100,000 MWCO filter. The virus titer was measured by infecting HEK293 cells and stored in -80 °C before use. For production of lentiviruses, briefly, the lentiviral expression shuttle vector and three helper plasmids (pRSV-REV, pMDLg/pRRE and vesicular stomatitis virus G protein (VSVG)) were co-transfected into HEK293T cells using calcium phosphate. In total, 12 µg of lentiviral packaging DNA were transfected per T75 flask of HEK293T cells: REV (4 µg), RRE (8 µg), and VSVG (6 µg). Transfections were performed using the calcium-phosphate method in media lacking antibiotics (DMEM + 10% FBS). Media with viruses was collected at 72 h after transfection, cell media was harvested and centrifuged at 23,000g for 2 h. Pellets were resuspended overnight at 4 °C in 100 µl of DMEM, aliquoted and frozen at -80 °C before use. Viral infectious titer was determined, and equal numbers of infectious particles were injected during each experiment.

Primary Mixed Neuron-Glia Cultures. The entorhinal cortices or hippocampi were dissected from newborn (DIV0) mice, digested with papain (Worthington) for 20 min at 37 °C, and filtered through a 70 mm cell strainer (Falcon). Cells were plated on Matrigel- (Corning) coated coverslips in 24-well plates. Plating media contained 5% fetal bovine serum (Sigma), B27 (Gibco), 0.4% glucose (Millipore-Sigma), 2 mM glutamine (Gibco) in MEM (Gibco). At DIV1, the culture medium was changed to growth medium containing B27 (Gibco), 2 mM glutamine (Gibco) in Neurobasal A (Gibco). At DIV3.5, half of the medium was exchanged for growth medium containing 4 mM Ara-C (Millipore-Sigma). Neurons were analyzed at DIV14-16 and DIV21.

Sparse Transfections. Entorhinal cortex neurons were sparsely transfected with tdTomato using a calcium phosphate method at DIV9 to achieve sparse delivery of plasmids to isolated neurons that could be identified by their tdTomato expression. A DNA/calcium phosphate precipitate was prepared by mixing the following (per well, 24 well plate): 1 µg of DNA, 1.75 µl of 2M $CaCl_2$, and ddH_2O to a volume of 15 µl. DNA mixture was added dropwise under constant, low-powered vortex to an equal volume of 2x HEPES-buffered saline (270 mM NaCl, 10 mM KCl, 1.5 mM Na_2HPO_4, 10 mM D-glucose, 40 mM HEPES, pH 7.05). The precipitate formed for 15 min at room temperature before addition to the cultures. Cultured neurons were washed three times using MEM and finally were placed in serum-free MEM (0.5 ml per well). The original conditioned media was saved. DNA/calcium phosphate precipitate (30 µl) was added dropwise to each well. Dishes were returned to 5% CO_2 incubator at 37 °C for 20 min. The incubation was stopped by washing the cells twice with 1 ml per well of MEM. The saved conditioned medium was added back to each well, and the cells were returned to CO_2 incubator and analyzed at DIV14-16.

Monosynaptic Retrograde Pseudo-Typed Rabies Tracing and Stereotactic Injections. For presynaptic trans-synaptic rabies virus tracing (*31*), newborn pups were anesthetized on ice for 3-

5 min and immobilized on an ice bag with ear bars. Injection coordinates were zeroed from lambda. 0.15 µl of 10^8 IU/mL titer AAVs expressing NLS-tdTomato-ΔCre or NLS-tdTomato-Cre mixed with lentiviruses expressing Tenm3 rescue constructs were injected unilaterally into the MEC of the newborn mice. At P21, 0.05 µl AAVs constructed from the AAV-TCB-mCherry-P2A-RG vector were then injected into the proximal CA1 region of the same mice anesthetized with Avertin (250 mg/kg). At P35, mice were again anesthetized by injection of Avertin (250 mg/kg), head-fixed using stereotaxic equipment, and 0.2 µl glycoprotein-deleted pseudotyped rabies virus (EnvA) produced by the Janelia Farm Viral Core Facility was injected into the proximal CA1. After all injections, mice were allowed to completely recover on a heating pad in a clean cage. At P41-42, mice were sacrificed, perfused and analyzed by imaging.

Immunocytochemistry. All solutions were made fresh and filtered via a 0.22 µm filter prior to starting experiments. Cells were washed once with PBS, fixed with 4% PFA, 4% sucrose and PBS for 20 min at 4 °C, washed three times with PBS, and permeabilized in 0.2% Triton X-100 and PBS for 5 min at room temperature. Surface staining was performed in unpermeabilized samples (without 0.2% Triton X-100). Cells were subsequently placed in blocking buffer containing 5% goat serum (Millipore-Sigma), 5% BSA and PBS for 1 h at room temperature, incubated with diluted primary antibodies (Rabbit anti-HA, Cell Signaling Technologies, #3724; Mouse anti-HA, Cell Signaling Technologies, #2367; Chicken anti-MAP2, EnCor Biotechnology Inc, #AB_2138173; Guinea pig anti-VGLUT1, Millipore, #AB5905; Rabbit anti-HOMER1, Millipore, #ABN37; Rabbit anti-Gephyrin, #AB5725; Guinea pig anti-vGAT, Synaptic Systems, #131004) in blocking buffer overnight at 4 °C, washed three times with PBS, incubated with diluted fluorescently-conjugated secondary antibodies (Goat anti-Chicken IgY (H+L) Cross-Adsorbed Secondary Antibody, Alexa Fluor™ Plus 488, Invitrogen, #A-32931; Goat anti-Guinea Pig IgG (H+L) Secondary Antibody, Alexa Fluor™ 546, Invitrogen, #A-11074; Goat anti-Rabbit IgG (H+L) Highly Cross-Adsorbed Secondary Antibody, Alexa Fluor 647, Invitrogen, #A-21245; Goat anti-Mouse IgG (H+L) Highly Cross-Adsorbed Secondary Antibody, Alexa Fluor™ 546, Invitrogen, #A-11030; Goat anti-Rabbit IgG (H+L) Cross-Adsorbed Secondary Antibody, Alexa Fluor™ 546, Invitrogen, #A-11010; Goat anti-Guinea Pig IgG (H+L) Highly Cross-Adsorbed Secondary Antibody, Alexa Fluor™ 647, Invitrogen, #A-21450) in blocking buffer for 2 h at room temperature, washed three times with PBS, and mounted on UltraClear microscope slides (Denville Scientific) using DAPI Fluoromount-G (Southern Biotech). For STED super resolution microscopy imaging, the same primary antibodies were used as before, secondary antibodies were raised in goat were conjugated to Abberior STAR RED, STAR ORANGE, and 460 L (Goat anti-Rabbit IgG STAR 460L, Abberior, #ST460L-1002; Goat anti-Guinea Pig IgG STAR Orange, Abberior, #STORANGE-1006; Goat anti-Guinea Pig IgG STAR Red, Abberior, #STRED-1006) and used at 1:1000 dilution.

Immunohistochemistry. Mice were anesthetized by isoflurane and perfused trans-cardially with ice cold PBS followed by ice cold 4% paraformaldehyde (by weight) in PBS. Brains were dissected, post-fixed in 4% paraformaldehyde overnight at 4 °C for tracing staining or no staining, washed three times with PBS, cryoprotected for 72 h in 30% sucrose at 4 °C. The brains were then embedded in Optimum Cutting Temperature (OCT, Tissue Tek) and stored at -80 °C until being sectioned. Horizontal sections (60 µm) were collected at -20 °C using a cryostat. The free-floating sections were collected and washed with PBS and blocked for 1 h in blocking buffer (4% BSA, 3% normal goat serum, 0.3% Triton-X100, 0.05% sodium azide, and PBS) at room temperature. The primary antibodies (Rabbit anti-HA, Cell Signaling Technologies, #3724) were diluted in blocking solution and brain sections were incubated with primary antibody overnight at 4 °C,

followed by three times washes in PBS and 2 h incubation with secondary antibodies (Goat anti-Rabbit IgG (H+L) Highly Cross-Adsorbed Secondary Antibody, Alexa Fluor 647, Invitrogen Cat#A-21245) diluted in blocking buffer at room temperature. Sections were labeled with DAPI (Millipore, Cat#10236276001) diluted into PBS for 20 min at room temperature, washed 3 x 15 min with PBS, and mounted on glass slides coated in 0.1% Triton-X100 and PBS, dried, and coverslipped with Fluoromount-G (Southern Biotech #010001).

Culture Electrophysiology. Electrophysiological recordings on cultured neurons were performed essentially as previously described (*59*), with slight modifications. Cells were held at −70 mV in whole-cell patch clamp configuration, taking into account the predicted liquid junction potential of solutions used below. Patch pipettes with a resistance of 3-5 MΩ were pulled from borosilicate glass capillaries (World Precision Instruments, Cat# TW150-4) using a PC-10 pipette puller (Narishige). A Multiclamp 700B amplifier and Digidata 1550A Low-Noise Data Acquisition Digitizer with Clampex 10 data acquisition software (Molecular Devices) were used to monitor synaptic currents, sampled at 4 kHz. The extracellular bath solution was composed of (in mM) 140 NaCl, 5 KCl, 2 $MgCl_2$, 2 $CaCl_2$, 10 D-glucose, 10 HEPES (pH 7.4, adjusted with NaOH and ~295 mOsm). To isolate Miniature Excitatory Postsynaptic Currents (mEPSCs), action potentials and inhibitory currents were pharmacologically suppressed by adding 1 μM tetrodotoxin and 50 μM picrotoxin (Tocris), respectively, to the extracellular bath solution. The pipette solution contained (in mM) 135 Cs-Methanesulfonate, 8 NaCl, 10 HEPES, 0.3 EGTA, 0.3 Na_2GTP, 2 MgATP, 7 phosphocreatine, 0.1 spermine, and 10 QX-314 (Tocris) (pH 7.3, adjusted with CsOH and 306 mOsm). Cells of visually similar shape and size were targeted. After a stable patch was established, passive cell characteristics (access resistance (R_a), input resistance (R_m) and capacitance (C_m)) were measured and then continuously monitored. Acquisition as well as analysis with Clampfit 10 software (Molecular Devices) was performed while blinded to the experimental conditions. A 750 Hz Gaussian lowpass filter was applied before analysis. mEPSCs were automatically detected in a template-based search and visually inspected for inclusion or rejection. At least 100 consecutive events or 3 minutes of continuous recording were analyzed. Traces that displayed one or multiple of the following criteria were considered not analyzable, compromised or atypical and were therefore excluded: (1) Noise >5 pA or unstable baseline, (2) R_a >15 MΩ (or increase > 20% of initial value during recording), R_m <70 MΩ or a holding current <-350 pA, (3) Mean event amplitude >30 pA (< 2% of cells), (4) abnormally high event frequency, recognized as an outlier with 99.9% confidence using ROUT (GraphPad Prism) (<1% of cells).

Quantitative RT-PCR. To determine the Tenm3 and Tenm4 knockout efficiency, mRNA was prepared from both Entorhinal cortex cultures and hippocampal cultures that had been infected with ΔCre or Cre lentiviruses at DIV3.5 and collected at DIV14. RNA was extracted using the Qiagen RNeasy kit according to the manufacturer's protocol (QIAGEN) and quantified using an ND-1000 spectrophotometer (NanoDrop, Thermo Scientific). Quantitative real-time PCR (qRT-PCR) was performed using the VeriQuest Probe One-Step RT-qPCR Master Mix (Affymetrix) based on the manufacturer's instructions, and reactions were carried out and quantified using a 7900HT Fast RT-qPCR instrument (Applied Biosystems). Expression levels were normalized to Actin-b as an internal control. The following PrimeTime qPCR Assays (IDT) were used (shown as gene, primer1, probe and primer2): Tenm3, GTAGGCGGAGAATAAACAGAGC, CTGAACAGTGGCGTCGTCCCT, GTCCTTGGCAGTAATGTACCA; Tenm4, GTTGTCCTGCAATGTCCCTA, TGGAAACCAGAAACCTAGGCAAGCA, GGACAACTGGCTACTCAACA; mouse Actb (Applied Biosystems; cat. no. 4352933).

SDS-PAGE and Immunoblotting. For more sensitive detection of Tenms expression levels, entorhinal cortex neurons were harvested in N-PER Neuronal Protein Extraction Reagent (Thermo Fisher Scientific) with cocktail protease inhibitors and prepared for standard SDS-PAGE. Samples were diluted in Laemmli sample buffer (final concentration: 1X) containing fresh DTT and heated to 95 °C for 5 min. For detection of the Tenm3 and Lphn3 signals in the sedimentation assay of reconstituted synaptic junctions, 10 µl of sample was centrifuged at 5,000 rpm in the Eppendorf 5417C centrifuge for 5 min. The supernatant was immediately removed, and the pellet was resuspended in 1XLaemmli sample buffer. Proteins were separated by SDS-PAGE using 4-20% MIDI Criterion TGX precast gels (Bio-Rad) at 80 V constant voltage for 2 h, proteins were transferred onto nitrocellulose transfer membrane using a Criterion Blotter (Bio-Rad) with plate electrodes in ice-cold transfer buffer (25.1 mM Tris, 192 mM glycine, 20% methanol) at 80V constant voltage for 1 h or stained in Coomassie G-250 blue. Membranes were blocked in 5% non-fat milk (Carnation) diluted in TBST for 1 h at room temperature. Membranes were then incubated with primary antibodies (Rabbit anti-HA, Cell Signaling Technologies, #3724; Rabbit anti-FLAG, Cell Signaling Technologies, #F7425) diluted in 5% non-fat milk/TBST overnight at 4 °C. Membranes were washed 3 times followed by incubation with secondary antibodies (IRDye 800CW Donkey anti-Rabbit, LI-COR, #926-32213). Immunoblotting and quantitative analysis were performed by Odyssey Infrared Imager CLX and software Image Studio 5.2.5 (LI-COR Biosciences).

Confocal Image Acquisition and Analysis. Serial confocal z-stack images were acquired using a Nikon confocal microscope (A1RSi) with 10x, 20x, 60x, and 100x objectives. Images were analyzed using NIS-Elements AR acquisition software. Laser intensities and acquisition settings were established for individual channels using optimal LUT settings and applied to entire experimental replicates. All staining processes and acquisition parameters were kept constant among different experiments. For synaptic puncta and morphology quantification, z-stack images were acquired at 0.2 µm intervals and ten consecutive sections with the highest signal were projected maximally. For phase separation analysis, a single section of imaging was acquired. The background subtraction was measured within each set of the experiments, and the same background threshold was applied to each set of experiments and puncta parameters were automatically obtained using the analysis software Nikon NIS-Elements and ImageJ. For rabies tracing quantification as described, briefly, horizontal sections (60 µm) were imaged using a 10x and 20x objective, four regions-of-interest were collected: the proximal CA1 (starter cells), ipsilateral CA3, ipsilateral medial entorhinal cortex (MEC), and contralateral CA3. Cells were counted using Nikon NIS-Elements AR acquisition software and ImageJ in each region-of-interest.

Calcium Imaging. Calcium imaging in entorhinal cortex neurons were performed as described (*60*). Cultured neurons were infected with lentiviruses expressing hSyn-NLS-tdTomato-ΔCre/Cre at DIV4, and lentiviruses expressing indicated Tenm3 rescue proteins at DIV6, and lentiviruses expressing hSyn-GCaMP6m at DIV7. Calcium imaging was performed at DIV14-15 using a Leica microscope at 37 °C under 5% CO_2. For potentiating synaptic transmission in cultured neurons, the cells were imaged at an ambient 4 mM $CaCl_2$ and 8 mM KCl concentration equilibrated in a HEPES-based buffer (129 mM NaCl, 25 mM HEPES-NaOH, 15 mM glucose, 1 mM $MgCl_2$, 10 µM glycine, adjust pH to 7.2 - 7.4) for 2 min. Images were analyzed by MATLAB.

STED Microscopy. STED super-resolution images and confocal comparison images were acquired using a Nikon Ti2-E microscope stand equipped with a STEDYCON confocal and STED module from Abberior Instruments, Inc (*61*). Excitation lasers included 488 nm (pulsed), 595 nm

(pulsed), and 640 nm (pulsed). A 775 nm STED depletion laser (pulsed) was used. Detection was performed using time-gated APDs. Images were acquired using a CFI PLAN APO LAMBDA 100X OIL objective (NA 1.45) with a piezoelectric focusing system. Immersion oil F was used for all imaging. For quantitative analysis, images were acquired using identical settings. Image settings were optimized to ensure that signal was acquired below saturation, with saturation levels indicated using the look-up table. Acquisition laser intensity was scaled ~1.5X higher for STED imaging and the depletion laser was set to enable 60 nm resolution in all channels. Pixel size was automatically determined based on the resolution of the acquired image. For STED acquisition, 15-line accumulations were used, the same size of interest regions were collected. For all images STEDYFOCUS was used to allow sequential confocal and STED imaging. A single optical plane was imaged, as there was no depletion laser in the z plane. For analysis, OBF files were exported from the STEDYCON acquisition software and opened in Hugyen's Essentials from Scientific Volume Imaging. All STED images were deconvolved to afford minimal improvement in resolution and exported as ids files. They were then opened in Nikon Elements and puncta were analyzed using the general analysis software module after converting to nd2 files. For analyzings the distribution of nanoclusters and vGluT1 or Homer1 localizations in fig. S2F, histograms were fitted with the following functions in Matlab: Gaussian: $f(x) = a1*\exp(-((x-b1)/c1)^2) + a2*\exp(-((x-b2)/c2)^2)$; other images were processed using ImageJ and Matlab.

Protein Expression and Purification. PSD and active zone scaffold proteins (PSD-95, GKAP, SynGAP, Shank3, Homer3, RIM1 and RIM-BP2) were purified as described (*34, 62*) with slight modifications. DNAs encoding the various proteins were generated using standard PCR-based methods and cloned into a vector containing an N-terminal Trx-His_6 or a His_6-affinity tag followed by an HRV 3C cutting site. All constructs were confirmed by DNA sequencing. Recombinant proteins were expressed in *Escherichia coli* BL21 (DE3) (Thermo Fisher, #EC0114) in LB medium at 16 °C overnight and protein expression was induced by 0.25 mM IPTG (final concentration) at OD_{600} between 0.6 and 0.8. Each recombinant protein was purified using a nickel-NTA agarose affinity column (Qiagen, #30210) followed by the cleavage with HRV 3C protease. Proteins were further cleaned by a final step size-exclusion chromatography (Superdex 200 or Superdex 75, Cytiva) with a column buffer containing 50 mM HEPES-NaOH, pH 7.8, 100 mM NaCl, 1 mM EDTA, 1 mM TCEP. For purifications of PSD-95 and Homer3, a mono Q ion-exchange chromatography (Cytiva) was added to remove DNA contamination and degraded proteins after 3C digestion. For GKAP which contains three GK binding repeats for binding to PSD-95, the first GK binding repeat was replaced by a DLS sequence (a phospho-mimicking mutation) to enhance its binding to PSD-95 (*38*). For Shank3 protein containing wild-type SAM domains with enhanced self-oligomerization (*63*), a GB1 tag was introduced to the N-terminal to stabilize the protein. Wild-type and mutant $Tenm3^{ICD}$ proteins were expressed using MBP-His_6-3C-$Tenm3^{ICD}$-GB1-Alfa plasmid and its derivatives. Proteins were expressed in *Escherichia coli* BL21 (DE3) in LB medium cultured at 20 °C overnight and protein expression was induced by 1 mM IPTG (final concentration) at an OD_{600} between 0.8 and 1.0. Trx-Lphn3-ICD was expressed in *Escherichia coli* BL21 (DE3) in LB medium cultured at 37 °C for 2 hours, protein expression was induced by 1 mM IPTG (final concentration) at an OD_{600} at 1.0. Each ICD protein was purified using a nickel-NTA agarose affinity column followed by size-exclusion chromatography (Superdex 200) with a column buffer containing 50 mM HEPES-NaOH, pH 7.8, 300 mM NaCl, 1 mM EDTA, 1 mM TCEP. $Tenm3^{ECD}$ protein encoded by the plasmid AlfaNb-$Tenm3^{ECD}$-HA-3C-His_{10} was expressed in Expi293F™ cells at 37 °C. In brief, 50 mL of Expi293F™ (Thermo Fisher, A14527) cells were grown to 3×10^6 cells/mL. 50 μg of cDNA was

transiently transfected into Expi293F™ cells using 100 μL GibcoTM ExpiFectamine™ 293 Reagent (Thermo Fisher, #A14525). Enhancers were added 18 h after transfection and cells were incubated for another 96 hours before harvesting the medium. Proteins were purified by nickel-NTA agarose affinity column followed by - size-exclusion chromatography (Superdex 200) with a column buffer containing 50 mM HEPES-NaOH, pH 7.8, 100 mM NaCl. Lphn3 protein was expressed in Expi293F™ cells at 37 °C. Cells were collected 72 h after ExpiFectamine transfection. Cell pellets were lysed in a buffer containing 20 mM Tris pH 7.5, 1 mM EDTA, 15% v/v glycerol, 1 mM PMSF, 1X cOmplete™ Protease Inhibitor Cocktail (Roche, #04693159001) for 1 h at 4 °C. Membranes were collected by centrifugation at 37,000g for 30 min and homogenized in 0.5% (w/v) lauryl maltose neopentyl glycol (LMNG, Anatrace, #NG310) and 0.1% (w/v) cholesteryl hemisuccinate (CHS, Anatrace, #CH210) in solubilization buffer (20 mM HEPES pH 7.5, 150 mM NaCl, 15% v/v glycerol, 1 mM PMSF, 1X cOmplete™). Insoluble material was removed by centrifugation at 37,000g for 30 min. Lphn3 in supernatant was incubated with an Anti-Flag M2 Affinity Resin (Sigma, #A2220) for 2 h at 4 °C. The resin was packed into a gravity flow column and washed with a wash buffer containing 20 mM HEPES pH 7.4, 150 mM NaCl, 0.075% (w/v) LMNG, 0.025% (w/v) GDN (Anatrace, #GDN101) and 0.01% (w/v) CHS. Protein was eluted by Flag-elution buffer (20 mM HEPES pH 7.5, 150 mM NaCl, 0.075% (w/v) LMNG, 0.025% (w/v) GDN, and 0.01% (w/v) CHS and 0.25 mg/mL Flag peptide). Eluent was concentrated and loaded into size-exclusion chromatography (Superdex 200) with a column buffer containing 20 mM HEPES pH 7.5, 150 mM NaCl, 0.00075% (w/v) LMNG, 0.00025% (w/v) GDN and 0.0001% (w/v) CHS.

Protein Labeling with Fluorescent Dyes. For amide labeling (for Lphn3, Tenm3^{ECD}, PSD and active zone scaffold proteins), purified proteins were exchanged into a $NaHCO_3$ buffer (100 mM $NaHCO_3$, pH 8.3, 300 mM NaCl and 1 mM EDTA) and concentrated to 5-10 mg/mL. Alexa-647 NHS ester (Invitrogen, #A20006), Alexa-594 NHS ester (Invitrogen, # A20004), and iFluor-488 (AAT Bioquest, #1023)/iFluor-546 (AAT Bioquest, #1048)/iFluor-405 NHS ester (AAT Bioquest, #1021) were dissolved in DMSO at stock solutions of 10 mg/mL. Each dye and the protein to be labeled were mixed at a molar ratio of 1:1 and reacted for 1 h at room temperature. Reactions were quenched by 200 mM Tris, pH 8.2. Fluorophores and other small molecules were removed from the proteins by passing the reaction mixture through a HiTrap desalting column in buffer containing 50 mM HEPES-NaOH, pH 7.8, 100 mM NaCl, and 1 mM EDTA. For Lphn3, the protein was labeled in same condition in buffer containing 100 mM $NaHCO_3$, pH 8.3, 100 mM, 0.00075% (w/v) LMNG, 0.00025% (w/v) GDN and 0.0001% (w/v) CHS. For cysteine labeling (for all Tenm3^{ICD} variants), Tenm3^{ICD} proteins were prepared in labeling buffer (50 mM HEPES-NaOH, pH 7.5, 300 mM NaCl, 1 mM EDTA and 1 mM TCEP) in a final concentration of 2 mg/mL. iFluor-488 maleimide (AAT, #1062) and STAR RED maleimide (Abberior, #STRED-0003-1MG) (stock solution with 10 mg/mL in DMSO) were added in a 1:5 protein-to-fluorophore molar ratio and incubated for 1 h at room temperature. The fluorophores and other small molecules were removed from the proteins by passing the reaction mixture through a HiTrap (Cytiva) desalting column in a buffer containing 50 mM HEPES-NaOH, pH 7.8, 300 mM NaCl, 1 mM EDTA and 1 mM TCEP. Fluorescence labeling efficiency was measured by Nanodrop. In imaging assays the fluorescently labeled proteins were diluted with the corresponding unlabeled proteins in the same buffer, with a final ratio of 1:50 for all proteins.

Phase Separation Assays. Imaging-based phase-separation assays were performed as described (*62*). Briefly, purified proteins (with affinity tags cleaved and removed; Fig. S11) were prepared in indicated buffer and pre-cleared via high-speed centrifugations (14,000 g for 10 min at 23 °C).

Proteins were then mixed or diluted with buffer to designated combinations and concentrations. Protein samples were injected into a homemade flow chamber for fluorescent imaging used with a Nikon confocal microscope and recorded at room temperature. Digital images were analyzed by the ImageJ software. Sedimentation-based phase-separation assays were typically performed in a final volume of each reaction of 50 µL. After 10 min equilibration at room temperature, protein samples were centrifuged at 14,000 g for 10 min at 23 °C on a table-top temperature-controlled micro-centrifuge. After centrifugation, the supernatant and pellet were immediately separated into two tubes. The pellet fraction was thoroughly re-suspended in the same buffer with the same volume as the supernatant fraction. Proteins from both fractions were analyzed by SDS-PAGE (4%-15% gradient gel) with Coomassie blue staining and immunoblotting as indicated. Band intensities were quantified using the ImageJ software.

Giant Unilamellar Vesicle (GUV) Generation. GUVs were prepared from lipid mixture composed of 86% 1-palmitoyl-2-oleoyl-glycero-3-phosphocholine (POPC), 10% 1-palmitoyl-2-oleoyl-sn-glycero-3-phospho-L-serine (POPS), 1% 1,2-dioctanoyl-sn-glycero-3-phospho-(1'-myo-inositol-4',5'-bisphosphate) (PI(4,5)P2), 2% 1,2-dioleoyl-sn-glycero-3-[(N-(5-amino-1-carboxypentyl) iminodiacetic acid)succinyl] (nickel salt) (DGS-NTA(Ni)), 1% 1,2-dipalmitoyl-sn-glycero-3-phosphoethanolamine-N-(biotinyl) (Biotinyl PE), 0.2% 1,2-dioleoyl-sn-glycero-3-phosphoethanolamine-N-(Cyanine 5) (Cy5 PE). Lipid mixture was dissolved in chloroform at 1mg/mL and stored at -20°C before use. GUV formation was assisted by Polyvinyl alcohol (PVA, MW ~145,000) hydration (*39*). In brief, PVA was dissolved in ddH_2O to a final concentration of 10% (m/v) at 95°C. 10 µl of PVA solution was dripped on a coverslip and manually spread into a uniform layer. The PVA-coated coverslips were dried at 37°C for 0.5 hour. 10 µl of lipid mixture was applied on the PVA surface and spread using a glass pipette. The dried glass slide was placed in a vacuum for at least 1 hour to remove residual chloroform. PVA coverslip coated with lipid was transferred to 24 plate well treated with 100 µl hydration buffer (300 mM sucrose, 20 mM Tris-HCl pH 7.8). The hydration was proceeded at room temperature for 1hour in the dark and the solution containing GUVs was withdrawn and kept at 4°C.

Active Zone Reconstitution on GUVs and Imaging Assays. GUV imaging assays were performed in 96-well optical plastic-bottom microplates pre-coated with neutravidin (0.7 mg/mL dissolved in PBS) at room temperature for 1 hour. 10 µl GUV solution was diluted in 90 µl PBS and loaded into imaging wells. Imaging plates were placed at room temperature for 1 hour to allow GUVs to sediment and immobilized on bottom surface. Immobilized GUVs were incubated with 2 µM His_6-tagged Tenm3-ICD at room temperature for 0.5 hour. The untethered Tenm3-ICD was washed away by imaging buffer (50 mM HEPES-NaOH, pH 7.8, 100 mM NaCl, 1 mM TCEP). To induce LLPS, 2 µM RIM1 and RIM-BP2 proteins were added into the imaging wells and the mixture was incubated at room temperature for 30 mins before confocal imaging.

Data Analyses and Statistics. All bar plots show means ± SEM. In all box plots, the lowest datapoint shows the minimum value, the highest datapoint the maximum value, the central line the median and the box the interquartile range (25th to 75th percentile). For all violin plots, the central line shows the median and the upper and lower lines the quartiles (25th and 75th percentile). Statistical analyses were performed with GraphPad Prism 10 software using unpaired two-tailed Student's t-tests for two groups, Kolmogorov–Smirnov test for cumulative distributions, one-way ANOVA with Tukey's multiple comparison tests to compare the mean of each column with the mean of every other column, or two-way ANOVA with Bonferroni's multiple comparison tests for multiple groups with two variables (***P < 0.001, **P < 0.01, *P < 0.05, non-significant

comparisons are not indicated). Statistical analyses of the mean mEPSC frequency, amplitude, and passive cell characteristics were performed using one-way ANOVA comparing all conditions with the ΔCre condition and with Dunnetts's multiple comparison tests. For all rescue experiments, the predefined statistical comparison used to define rescue vs. non-rescue was a comparison of all groups to the ΔCre controls. All numbers of sections, neurons, mice, and replicates analyzed are shown in the bars or plots. All experiments were performed and analyzed in a blind manner by an experimenter except for the immunoblots for which this is not possible. All figures and data were scanned by 'Proofig' software to avoid accidental copy-paste errors in the assembly of figures, tables, and graphs.

Data Deposition. All raw data for this paper are publicly available at Stanford Digital Repository (https://purl.stanford.edu/xx501gx1493).

SUPPLEMENTARY FIGURES and LEGENDS

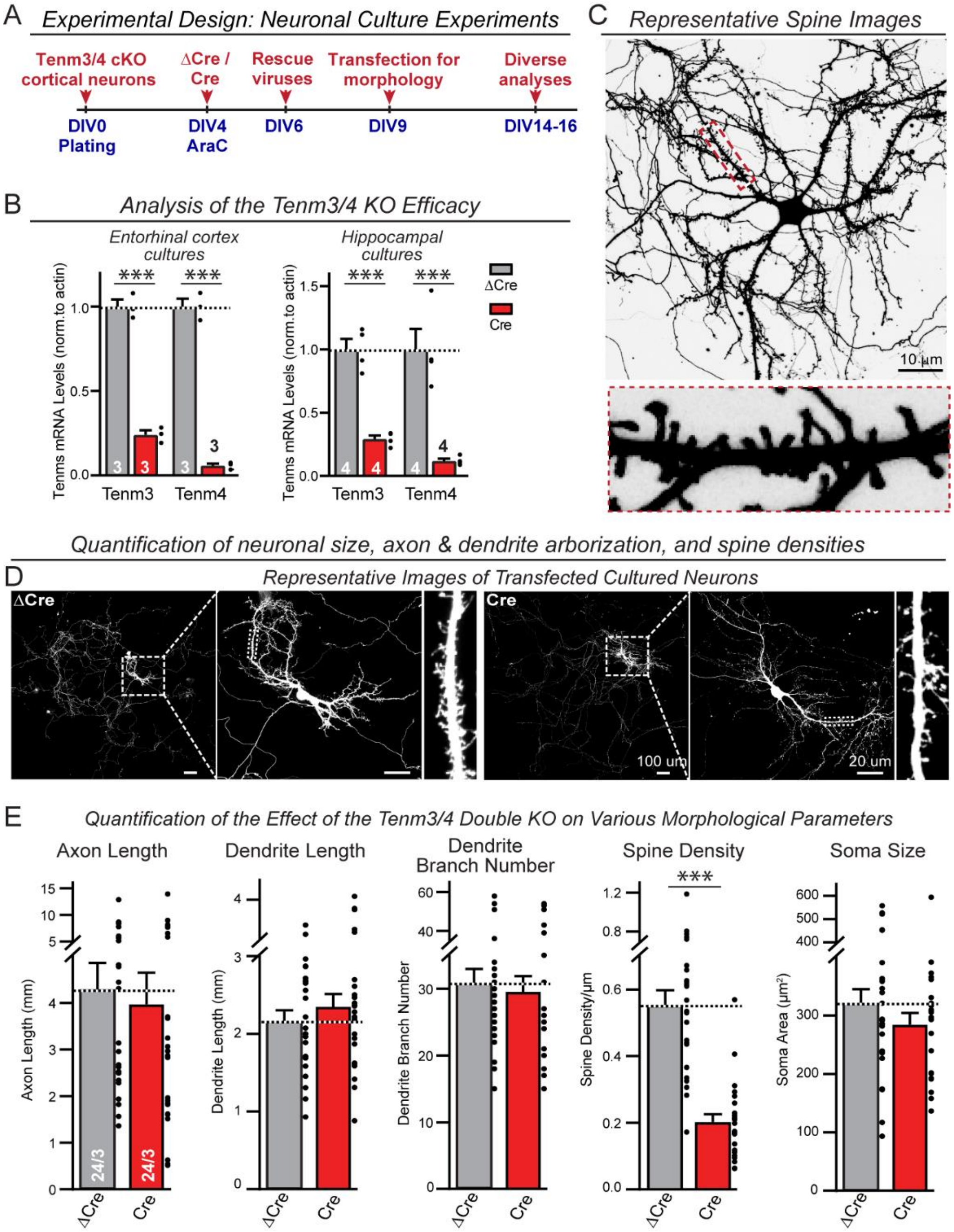

Fig. S1. Double deletion of Tenm3/4 in neuron-glia cultures from newborn Tenm3/4 cKO mice decreases the dendritic spine density without altering the sizes of axons, dendrites, or neuronal somata

(**A**) Experimental strategy for all neuronal culture experiments. Mixed entorhinal cortex or hippocampal neuron-glia cultures obtained from newborn Tenm3/4 cKO mice were infected at DIV4 with lentiviruses expressing active (Cre) or inactive mutant Cre-recombinase (ΔCre), both fused to EGFP or to tdTomato and containing a nuclear localization signal (*64*) and driven by the human synapsin promoter (hSyn-nls-EGFP/tdTomato-Cre and hSyn-nls-EGFP/tdTomato-ΔCre,

respectively). Cre- or ΔCre-expression in all neurons was confirmed via the nuclear EGFP or tdTomato signal. For rescue experiments, neurons were additionally infected with rescue lentiviruses at DIV6; for morphological axon and dendrite tracings, neurons were additionally sparsely transfected with tdTomato for at DIV9. Neurons were analyzed at DIV14-16.

(**B**) qRT-PCR analysis of Tenm3 and Tenm4 mRNA levels in entorhinal cortex and hippocampal cultures infected with control (ΔCre) and Cre-recombinase expressing lentiviruses (Cre).

(**C**) Representative image of the tdTomato signal in a sparsely transfected neuron to document the presence of abundant dendritic spines at DIV14.

(**D**) Representative images of the tdTomato signal in sparsely transfected Tenm3/4 double conditional knockout neurons expressing Cre or ΔCre to illustrate the approach used to measure axonal and dendritic arborizations, dendritic spine densities, and soma sizes at DIV14.

(**E**) Summary graphs of the axon and dendrite lengths, dendritic branch numbers, spine densities, and somatic areas as a function of the double deletion of Tenm3 and Tenm4. The remaining spines and synapses after the Tenm3/4 deletion are likely dependent on other, redundant synaptic adhesion molecules, such as Tenm1 and Tenm2 that are still expressed after the Tenm3/4 deletion or EphB receptors, neurexins, and LAR-type phosphotyrosine phosphatase receptors (*52-54*). In addition, deletion of intracellular planar cell polarity genes causes a similar decrease in synapse numbers (*65*).

Numerical data in B and E are means ± SEM (numbers of cells and experiments are indicated in bars), with ****P* < 0.001, ***P* < 0.01, **P* < 0.05 [B: Two-way ANOVA with post-hoc Tukey tests; E: two-tailed t test].

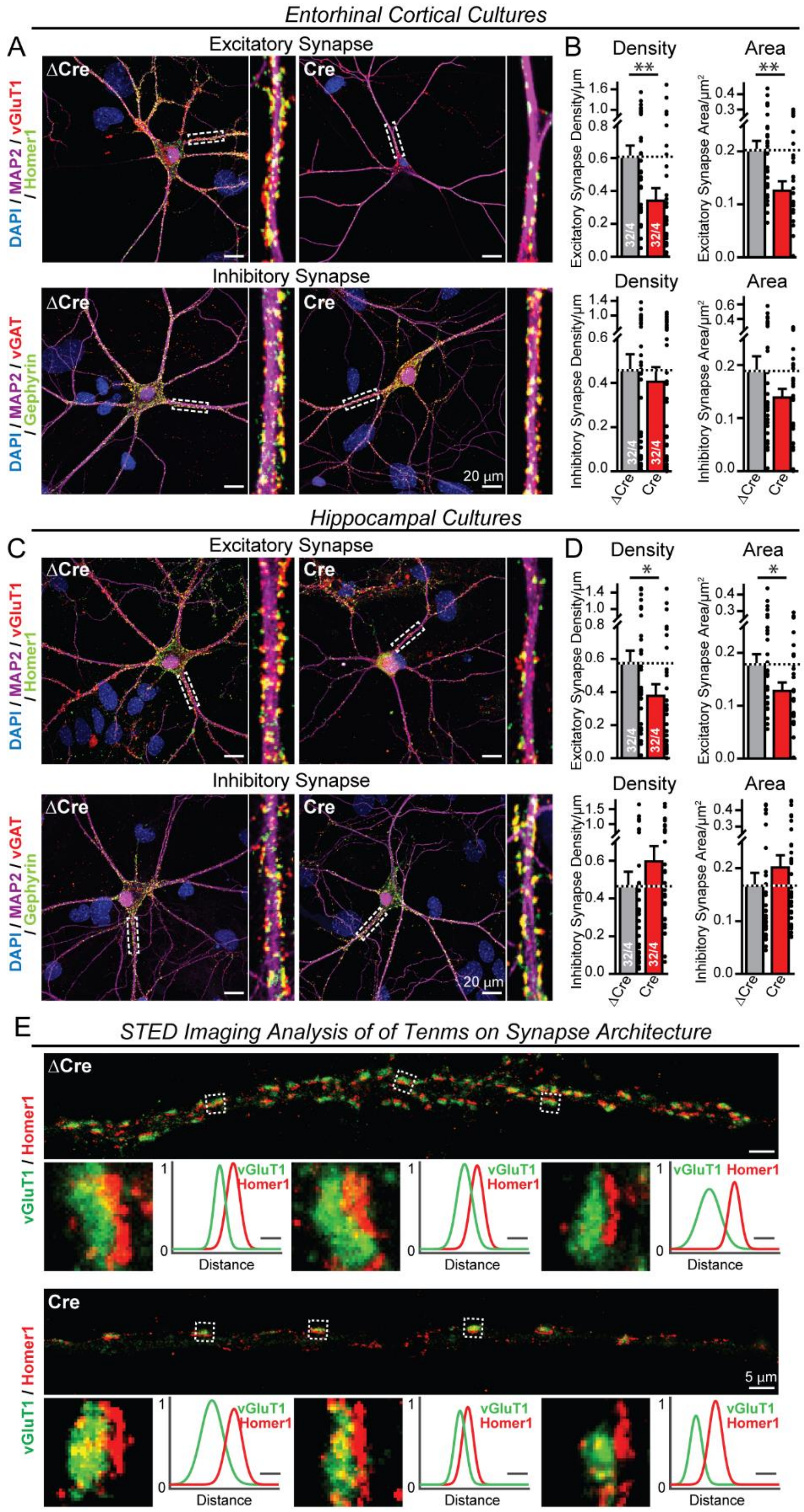

Fig. S2. Double deletion of Tenm3/4 in entorhinal cortex or hippocampal neuron-glia cultures selectively decreases the apparent density and sizes of excitatory but not inhibitory synapses

(**A** to **D**) Summary graphs of the excitatory and inhibitory synapse density and sizes in cultures from the entorhinal cortex (**A** and **B**) or hippocampus (**C** and **D**) (**A** and **C**, representative images of neurons stained for vGluT1/Homer1 (excitatory synapse puncta), vGAT/Gephyrin (inhibitory synapse puncta), MAP2, and DAPI (left, low magnification images; right, higher-magnification images taken from the boxed areas in the left images; **B** and **D**, summary graphs of the synapse density and size of excitatory (top) and inhibitory synapses (bottom)). For quantifications, synapses were defined as puncta imaged with identical parameters that are positive for both vGluT1 and Homer1 signals (excitatory synapses) or vGAT and gephyrin signals (inhibitory synapses). Data are means ± SEM (numbers of cells and experiments are indicated in bars), with ***$P < 0.001$, **$P < 0.01$, *$P < 0.05$ [two-tailed t test]. The absolute amount of synapse loss induced by the Tenm3/4 deletion differs between experiments owing to variations in virus preparations, precise media composition, culture handling, and other intangible experimental circumstances, which is why each experiment includes a positive and negative control.

(**E**) Representative STED images and signal traces showing that deletions of Tenm3/4 in cultured entorhinal cortex neurons do not detectably alter the width of the remaining synapses. Neurons were stained for vGluT1 (green) and Homer1 (red) (top, overview of dendritic segments; bottom, three examples of synapses with the vGluT1 and Homer1 signal shown on the left and a graph of the distribution of the vGluT1 and Homer1 signals along the distance from the center of mass on the right).

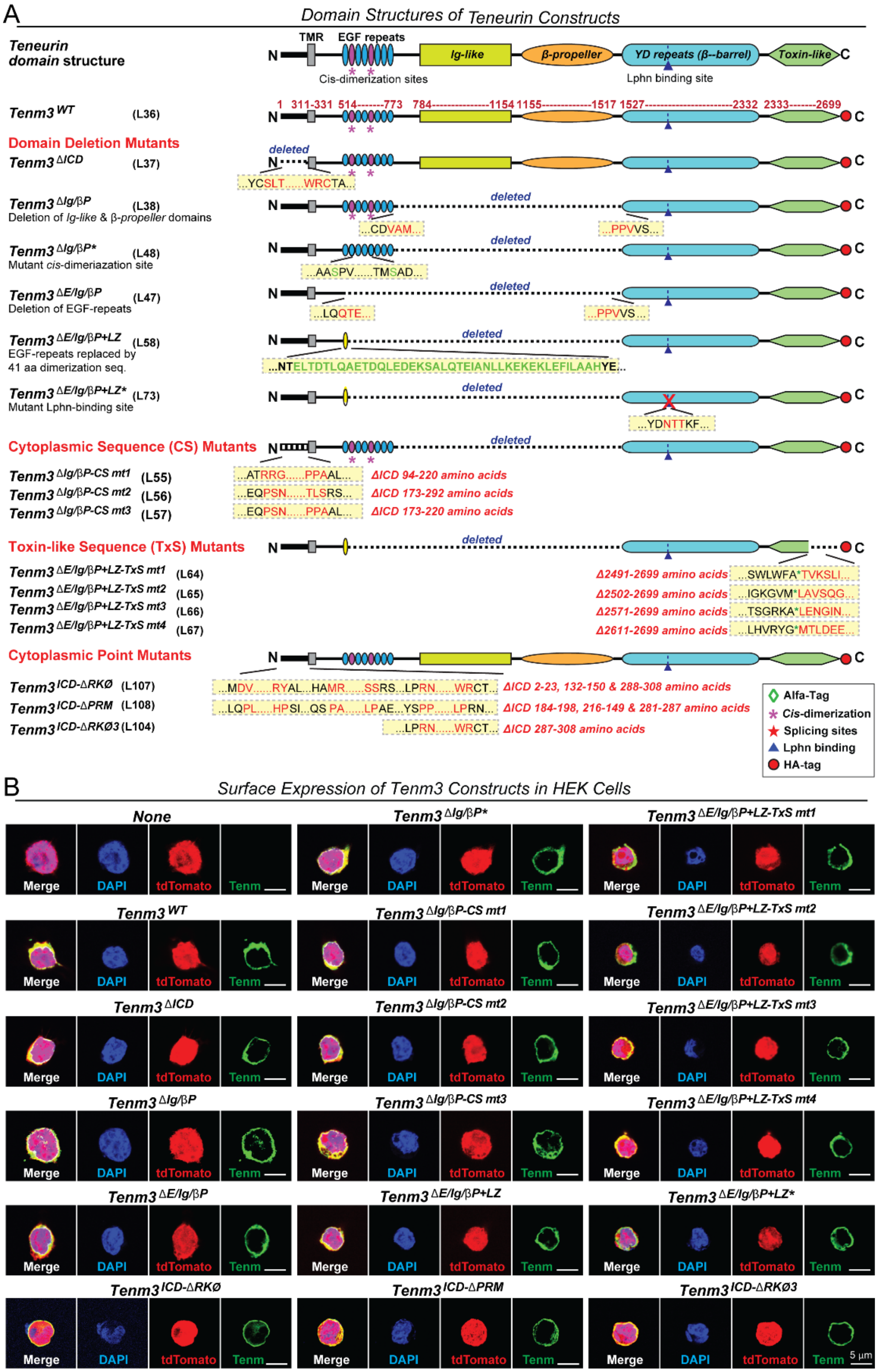

Fig. S3. Domain structures and surface expression of wild-type (WT) and mutant Tenm3 proteins encoded by various rescue constructs

(**A**) Schematic of the domain structures of wild-type (WT) and mutant Tenm3 proteins used for rescue experiments. The 'L36' to 'L108' tags refer to lab plasmid designations.

(**B**) Representative images of transfected HEK293T cells expressing the indicated wild-type and mutant Tenm3 proteins and stained for surface-exposed Tenm3 proteins via their C-terminal HA-epitope tags (see panel A). Proteins were co-expressed with tdTomato, unpermeabilized transfected HEK293T cells were surface-labeled for the HA-epitope prior to DAPI-staining, and cells were imaged by confocal microscopy.

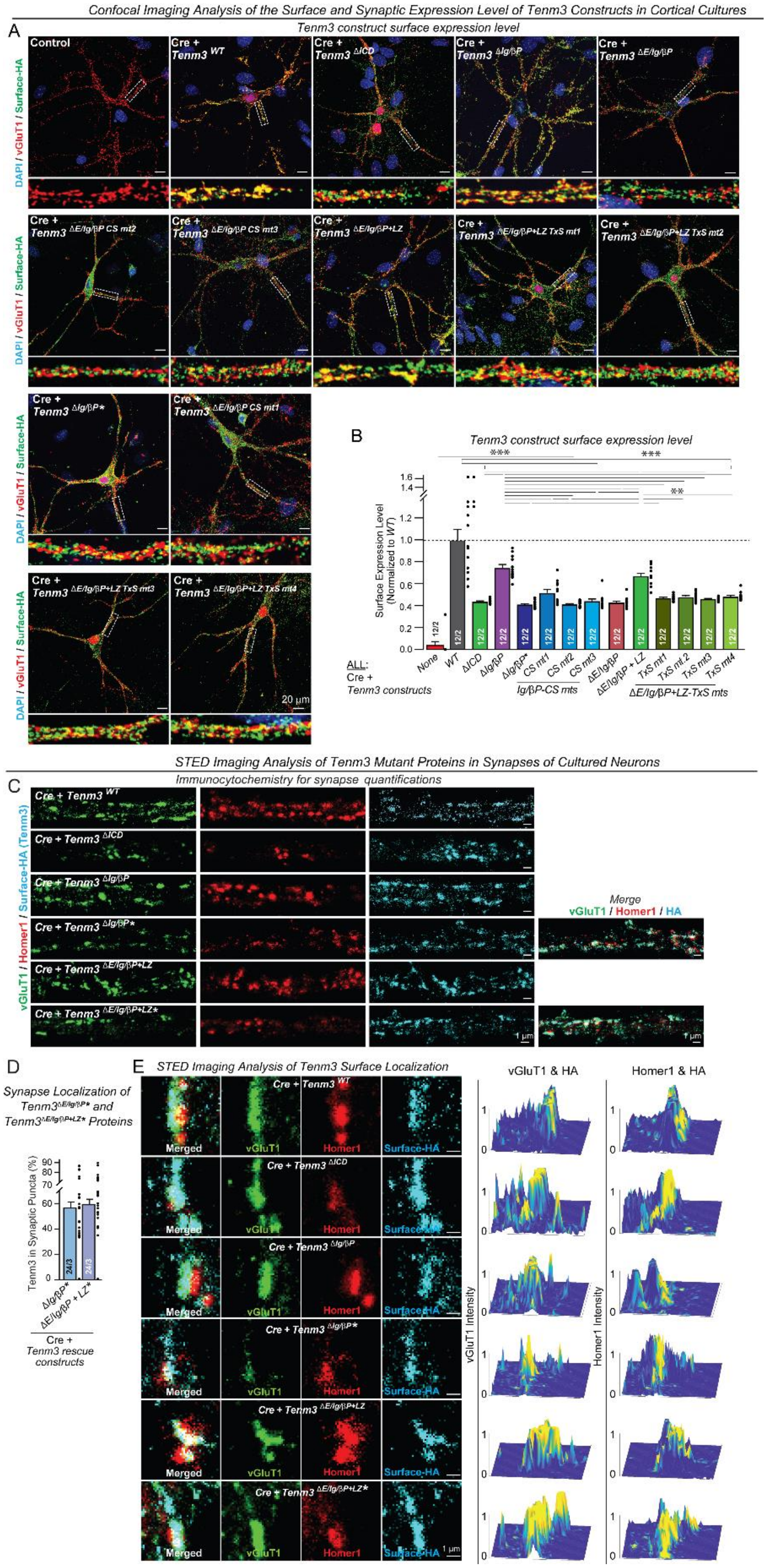

Fig. S4. Surface expression levels and synapse localization of wild-type (WT) and mutant Tenm3 proteins in cultured entorhinal cortex neurons

(**A**) Representative images of entorhinal cortex neurons infected at DIV4 with lentiviruses expressing nuclear tdTomato-tagged Cre recombinase (hSyn-nls-tdTomato-Cre) and at DIV6 with lentiviruses encoding the indicated WT and mutant Tenm3 proteins. Neurons were immunostained at DIV14-15 for surface HA to detect expressed Tenm3 proteins, vGluT1, and DAPI. Higher-magnification images (bottom) were taken from the boxed areas shown in the corresponding lower-magnification images (top).

(**B**) Quantification of the surface expression levels of WT and mutant Tenm3 proteins measured as the surface HA-signal normalized to the WT Tenm3 control signal using identical confocal settings in the same imaging sessions.

(**C** and **D**) Single-channel images from STED super-resolution microscopy experiments (Fig. 1B and C) and additional STED super-resolution data for the two constructs encoding mutant versions of the constructs analyzed in Fig. 1B and C (these mutants were not included in the main figures) (C, representative images; D, summary graphs of the fraction of HA-tagged Tenm3 proteins that are localized to synapses for the two mutant constructs not included in Fig. 1B and C).

(**E**) STED imaging analysis of individual synapses from the experimental series shown in C (left, representative STED images; right, 3D rendering heatmaps of the HA-Tenm3 signal in comparison to the vGluT1 and Homer1 signal intensities based on STED imaging).

Numerical data (B, D, and G) are means ± SEM (numbers of cells and experiments are indicated in bars); ***$P < 0.001$, **$P < 0.01$, *$P < 0.05$ [one-way ANOVA with post-hoc Tukey tests].

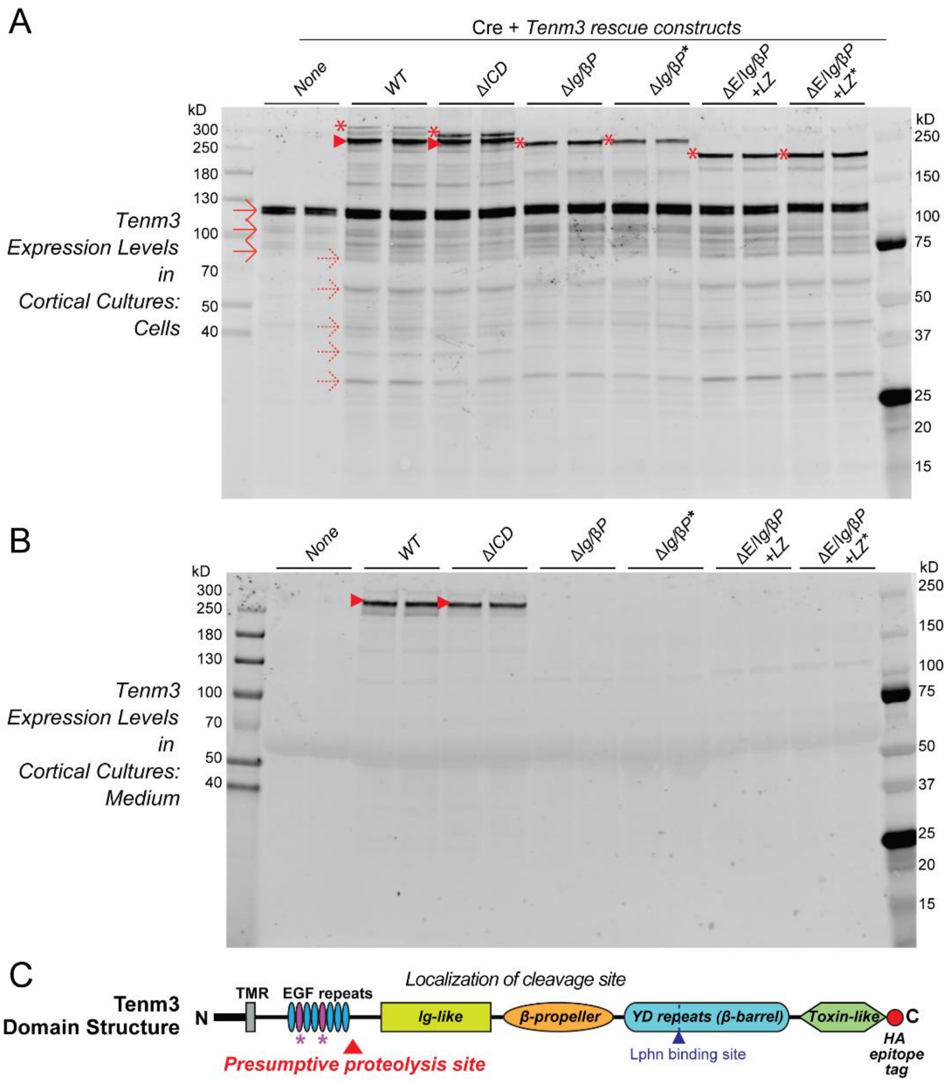

Fig. S5. Immunoblotting analysis of the expression and proteolytic processing of Tenm3 constructs in cultured entorhinal cortex neurons

(**A** and **B**) Immunoblots of WT and mutant HA-tagged Tenm3 proteins expressed in entorhinal cortex neurons (A, cells; B, medium). Cultured Tenm3/4 double cKO neurons were infected at DIV4 with lentiviruses expressing Cre and at DIV6 with lentiviruses expressing the indicated rescue proteins (none = no rescue protein expression as a negative control). The cells and media were analyzed at DIV14-15 (asterisks, WT and mutant Tenm3 proteins; arrowheads, proteolysis product secreted into the medium; solid arrows, non-specific bands; dashed arrows, HA-Tenm3 breakdown products). Note that no small fragment corresponding to TCAP was detected in the cells or media even though the C-terminal HA-tag should have made it visible (n = 2).

(**C**) Localization of the presumptive proteolysis site in the Tenm3 domain structure based on the size of the secreted cleavage product (>250 kDa) and the abolition of cleavage when the Ig-like domain is deleted.

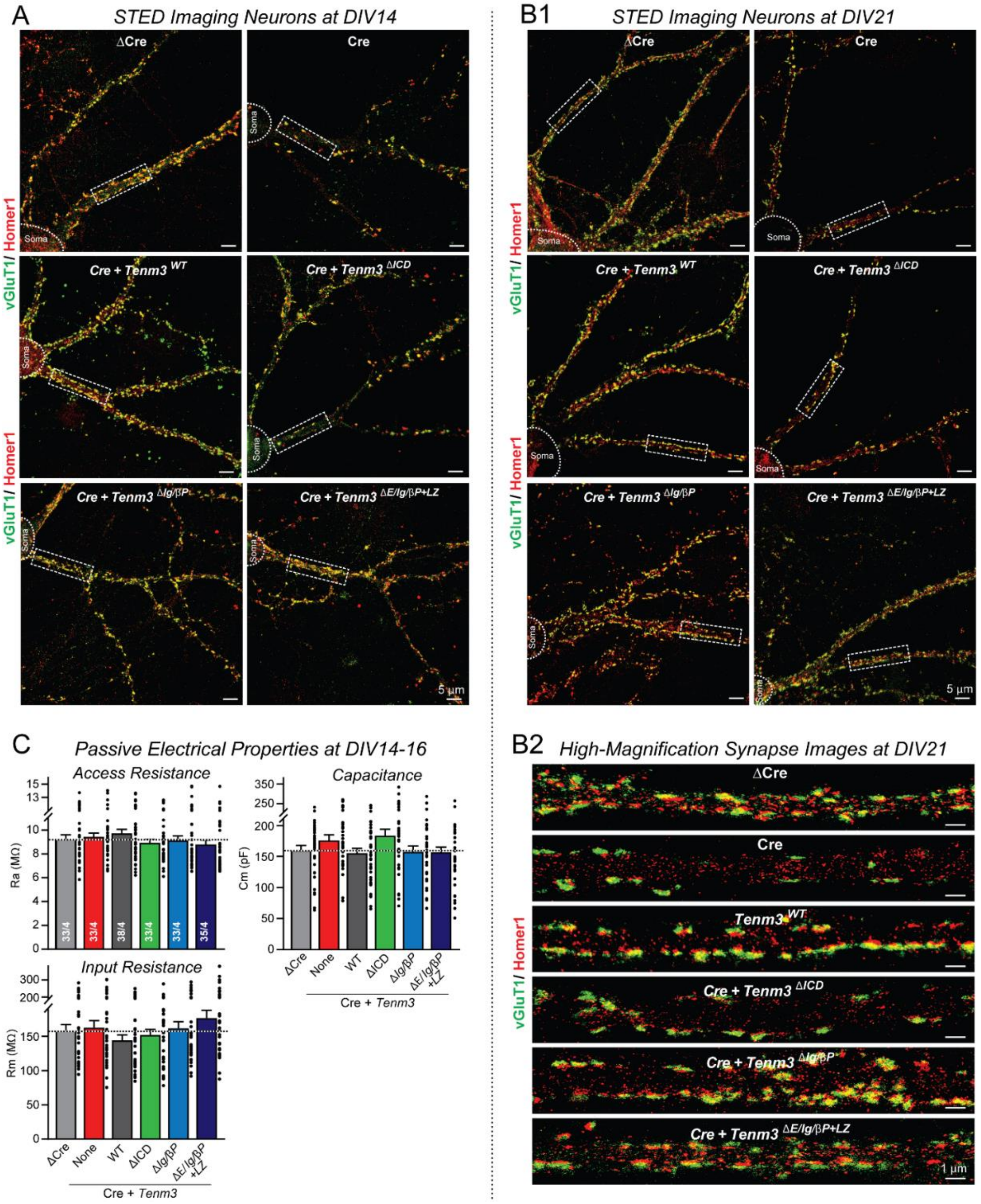

Fig. S6. Additional data complementing the STED super-resolution microscopy results in Fig. 1D and E and the electrophysiology results in Fig. 1F to H

(**A**) Representative lower magnification STED images corresponding to Fig. 1D at DIV14.

(**B**) Representative lower (B1) and higher magnification (B2) STED images corresponding to Fig. 1E at DIV21.

(**C**) Summary graphs of the neuronal passive electrical properties (access resistance, capacitance, input resistance) monitored in the electrophysiology experiments of Fig. 1. Data are means ± SEM (numbers of cells and experiments are indicated in bars), with no statistically significant difference among measured values [one-way ANOVA with post-hoc Dunnett tests].

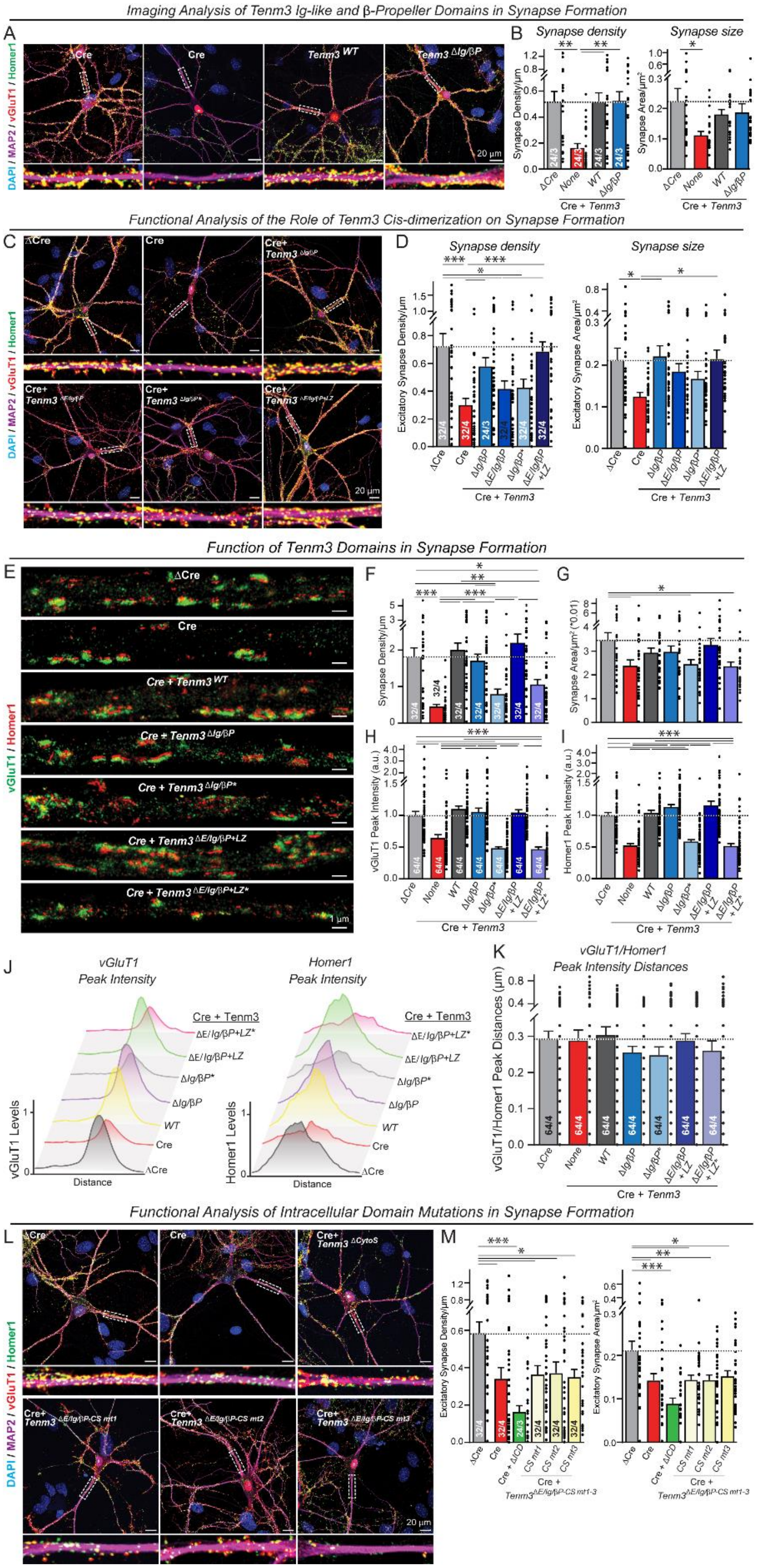

Fig. S7. Additional experiments analyzing rescue of synapse loss in Tenm3/4 double-deficient neurons by expression of various Tenm3 mutant rescue constructs. All experiments were

performed in cultured entorhinal cortex neurons immunostained for vGluT1 and Homer1 (to monitor excitatory synapses), MAP2 (to monitor dendrites), and DAPI (to stain nuclei) using the timelines shown in fig. S1A and the constructs described in fig. S3. As always, synaptic puncta are defined as puncta that are positive for both vGluT1 and Homer1.

(**A** and **B**) Rescue of synapse numbers in Tenm3/4-deficient neurons by Tenm3 lacking the Ig-like and β-propeller domains that are essential for trans-homodimerization (A, representative confocal images of immunostained neurons; B, summary graphs of the synapse density and size).

(**C** and **D**) Deletion of the Tenm3 EGF-like domains and point mutations in the *Cis*-dimerization sequences of the EGF-like domains abolish rescue of the synapse loss induced by the Tenm3/4 deletion, but rescue can be recovered upon addition of an artificial 41-residue dimerizing leucine zipper sequence (C, representative images; D, summary graphs of the synapse density and size).

(**E** to **I**) STED super-resolution imaging demonstrating that rescue of synapse numbers in Tenm3/4 double-deficient neurons by the minimal Tenm3 proteins requires latrophilin-binding. Experiments compare the rescue effect of $Tenm3^{\Delta E/Ig/\beta P+LZ}$ vs. $Tenm3^{\Delta E/Ig/\beta P+LZ}$* proteins that only differ by a point mutation abolishing latrophilin binding (see fig. S3) (E, representative images; F-I, summary graphs of the synapse density, size, vGluT1- and Homer1-staining intensity, respectively).

(**J**) The averaged normalized vGluT1 and Homer1 peak intensity profile across synapses expressing wild-type (WT) and mutant Tenm3 proteins determined in the experiments of E, H, and I. Note that the Tenm3/4 deletion in cultured entorhinal cortex neurons decreases the vGluT1 and Homer1 peak intensity in a manner that is rescued by $Tenm3^{WT}$, $Tenm3^{\Delta Ig/\beta P}$, and $Tenm3^{\Delta E/Ig/\beta P+LZ}$ but not by $Tenm3^{\Delta Ig/\beta P*}$ and $Tenm3^{\Delta E/Ig/\beta P+LZ*}$.

(**K**) Summary graph of the distance between the peak vGluT1 and Homer1 signal intensity at synapses as a function of the Tenm3/4 deletion and expression of WT and mutant Tenm3 rescue proteins, revealing that none of the Tenm3 manipulations produced a significant change in the apparent width of a synapse. Experiments were described in Fig. 1 which analyzed entorhinal cortex neurons using STED super-resolution microscopy.

(**L** and **M**) Functional analysis of the effects of various deletions from the Tenm3 ICD (i.e., cytoplasmic sequences; see fig. S3) demonstrate that the entire intracellular domain is essential for synapse formation (J, representative images; K, summary graphs of the synapse density and size).

Numerical data are means ± SEM (numbers of cells and experiments are indicated in bars), with ***$P < 0.001$, **$P < 0.01$, *$P < 0.05$ [one-way ANOVA with post-hoc Tukey tests].

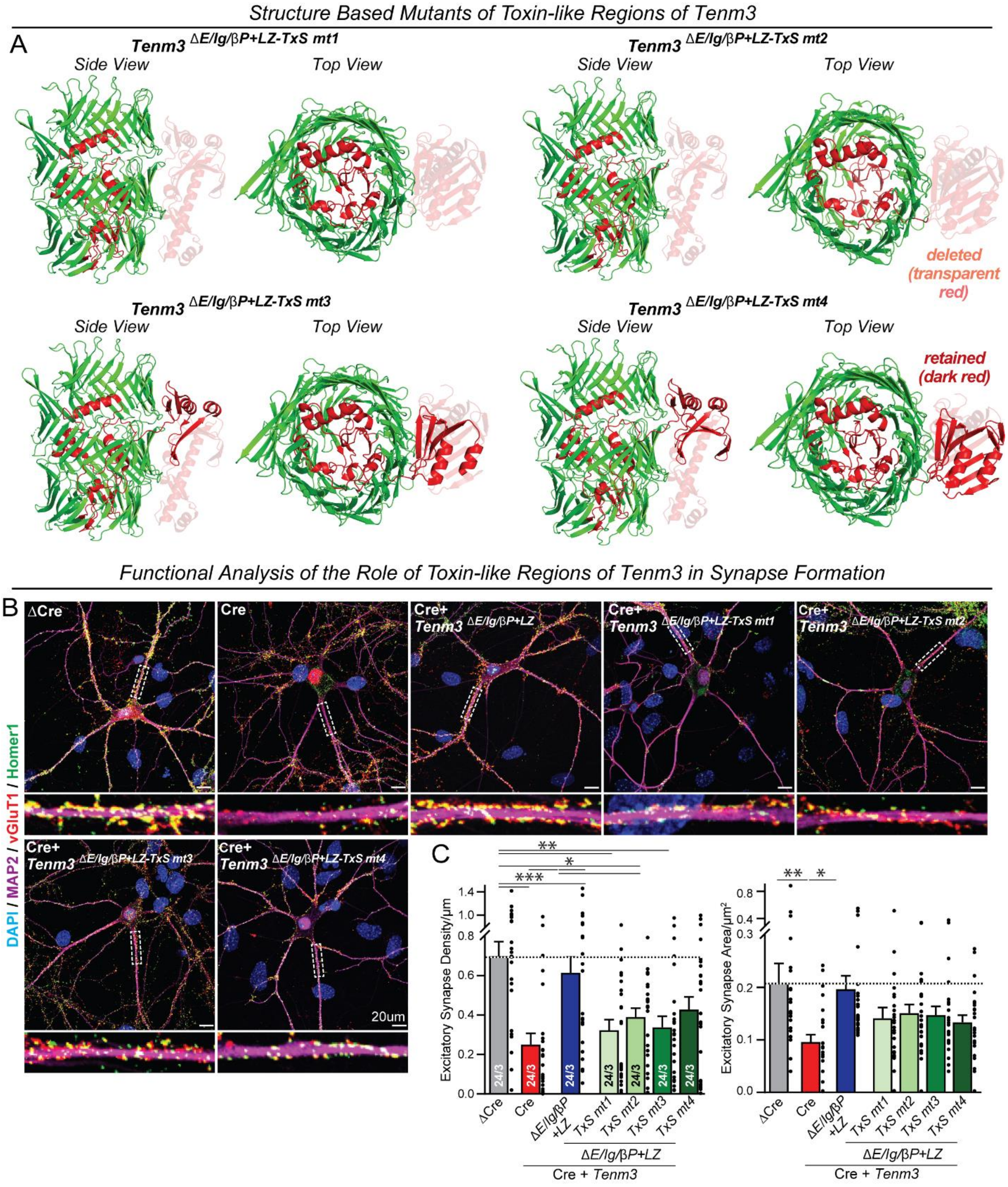

Fig. S8. Functional analyses reveal that the extracellular C-terminal toxin-like domain of Tenm3 is essential for synapse formation

(**A**) Modeling of the atomic structures of the C-terminal domains of mutant Tenm3 with various C-terminal deletions based on the Cryo-EM structures of teneurins (*10*) (green = β-barrel domain; red = toxin-like domain, with light red showing deleted and dark red showing retained sequences). For each mutant, a side view is shown on the left and a top view on the right.

(**B** and **C**) Functional analyses of the effects of various deletions from the toxin-like domain of Tenm3 document that the entire toxin-like region is essential for synapse formation (B, representative images; C, summary graphs of the synapse density and size). Data were obtained as described for fig. S7. Data in C are means ± SEM (numbers of cells and experiments are indicated in bars), with ***P < 0.001, **P < 0.01, *P < 0.05 [one-way ANOVA with post-hoc Tukey tests].

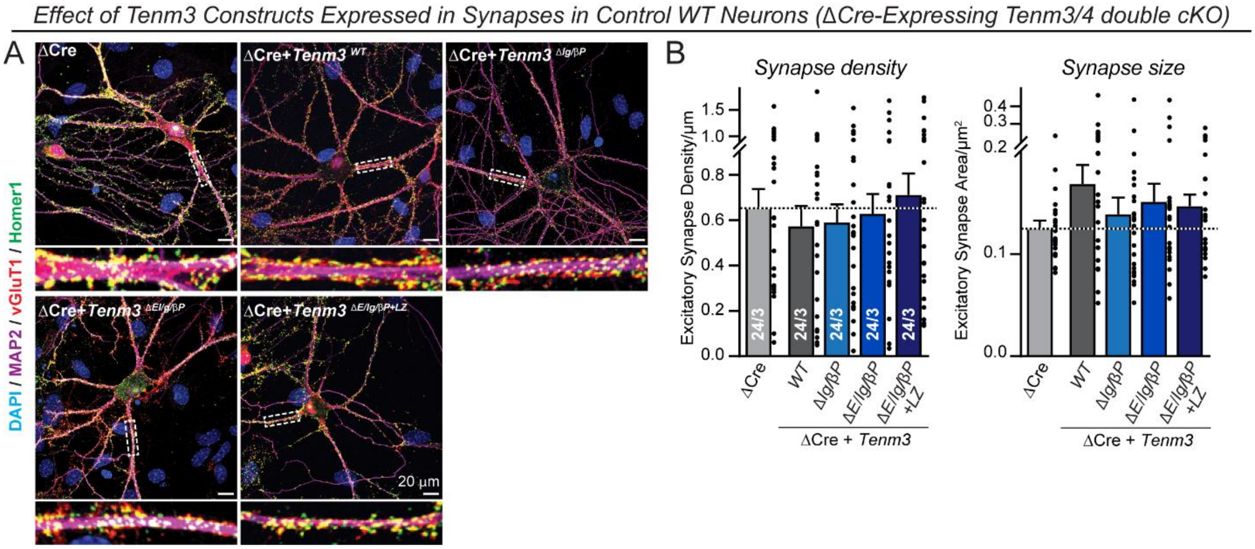

Fig. S9. Overexpression of wild-type (WT) and mutant Tenm3 proteins in neurons expressing normal levels of endogenous Tenm3 and Tenm4 has no effect on synapse numbers and size

(**A** and **B**) Effect of the overexpression of wild-type (WT) and mutant Tenm3 proteins in neurons expressing normal levels of endogenous Tenm3 and Tenm4 (**A**, representative images of confocal images of immune-stained neurons during measurements of synapse density; **B**, summary graphs of the synapse density and size).

Numerical data are means ± SEM (numbers of cells and experiments are indicated in bars), with ****P* < 0.001, ***P* < 0.01, **P* < 0.05 [one-way ANOVA with post-hoc Tukey tests].

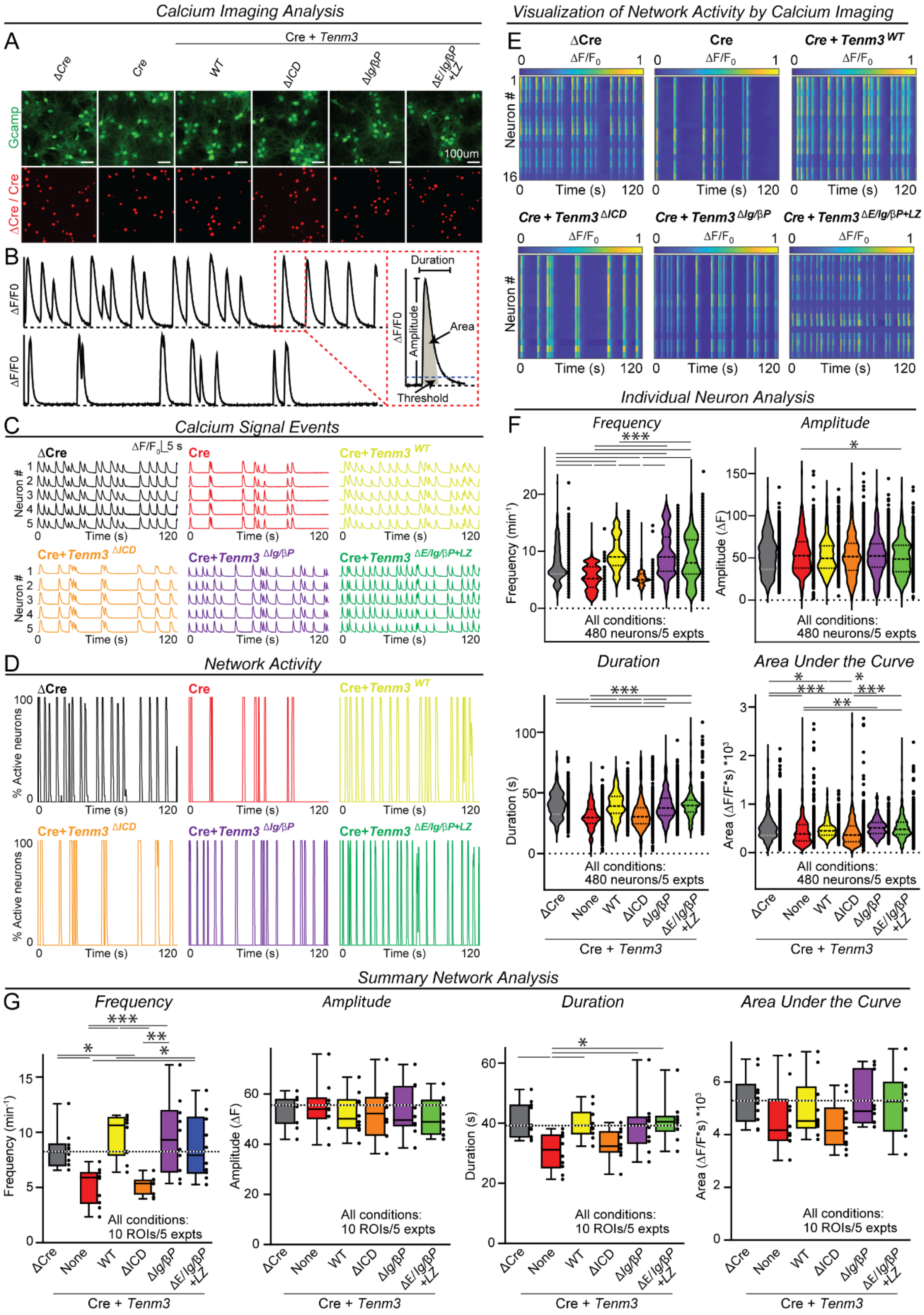

Fig. S10. Deletion of Tenm3/4 suppresses neural network activity as measured by Ca^{2+}-imaging in entorhinal cortex neurons in a manner that can be fully rescued by a Tenm3

protein containing only the N-terminal Tenm3 ICD and TMR, an exogenous short dimerization sequence (LZ), and the C-terminal Tenm3 β-barrel and toxin-like domains. The conditions and constructs used here are the same as in Fig. 1 as an independent validation approach.

(**A**) Representative Ca^{2+}-imaging experiment of cultured entorhinal cortex neurons from Tenm3/4 double cKO neurons expressing ΔCre or Cre tagged with nuclear tdTomato (red), GCaMP6m (green), and wild-type (WT) or mutant Tenm3.

(**B**) Illustration of the extraction of GCaMP6m signals (Ca^{2+}-imaging traces) from individual neurons in ΔCre (top) and Cre (bottom) groups (boxed area, detailed explanation of how the amplitude, duration, and area under the curve were quantified).

(**C** and **D**) Representative illustration of the extraction of GCaMP6m signals (Ca^{2+}-imaging traces) from individual neurons (C) and of the monitoring of the synchronous firing rate (D) in control neurons (ΔCre), Tenm3/4-deficient neurons (Cre) and Tenm3/4-deficient neurons expressing wild-type ($Tenm3^{WT}$) and mutant Tenm3 constructs ($Tenm3^{\Delta ICD}$, $Tenm3^{\Delta Ig/\beta}$, and $Tenm3^{\Delta E/Ig/\beta P+LZ}$).

(**E**) Representative heatmaps of GCaMP6m-monitored Ca^{2+}-signals ($\Delta F/F_0$) in individual cultured entorhinal cortex neurons expressing various Tenm3 rescue proteins as described in Fig. 1.

(**F**) Quantification of the synchronous firing frequency (top left), amplitude (top right), duration (bottom left), and area under the curve (bottom right) from individual neurons in the Ca^{2+}-imaging experiments. Deletions of both Tenm3/4 decrease the firing frequency, duration, and area under the curve in cultured entorhinal cortex neurons. $Tenm3^{WT}$, $Tenm3^{\Delta Ig/\beta P}$, and $Tenm3^{\Delta E/Ig/\beta P+LZ}$ efficiently rescue the decrease in firing frequency, duration, and area under the curve induced by the Tenm3/4 deletion whereas $Tenm3^{\Delta ICD}$ does not rescue. Note that the Tenm3/4 deletion and the various rescue constructs have no major effect on the amplitude.

(**G**) Quantification of amplitude (top left), duration (top right), and area under the curve (bottom left) of the network activity monitored by Ca^{2+}-imaging. Deletions of both Tenm3/4 decrease the frequency and duration in cultured entorhinal cortex neurons. $Tenm3^{WT}$, $Tenm3^{\Delta Ig/\beta P}$, and $Tenm3^{\Delta E/Ig/\beta P+LZ}$ efficiently rescue the decrease of frequency and duration induced by the Tenm3/4 deletion, whereas $Tenm3^{\Delta ICD}$ cannot rescue. Manipulations of Tenm3 have no effect on the amplitude and area under the curve in the network activity.

Numerical data are means ± SEM (numbers of cells and experiments are indicated in bars), with $***P < 0.001$, $**P < 0.01$, $*P < 0.05$ [one-way ANOVA with post-hoc Tukey tests].

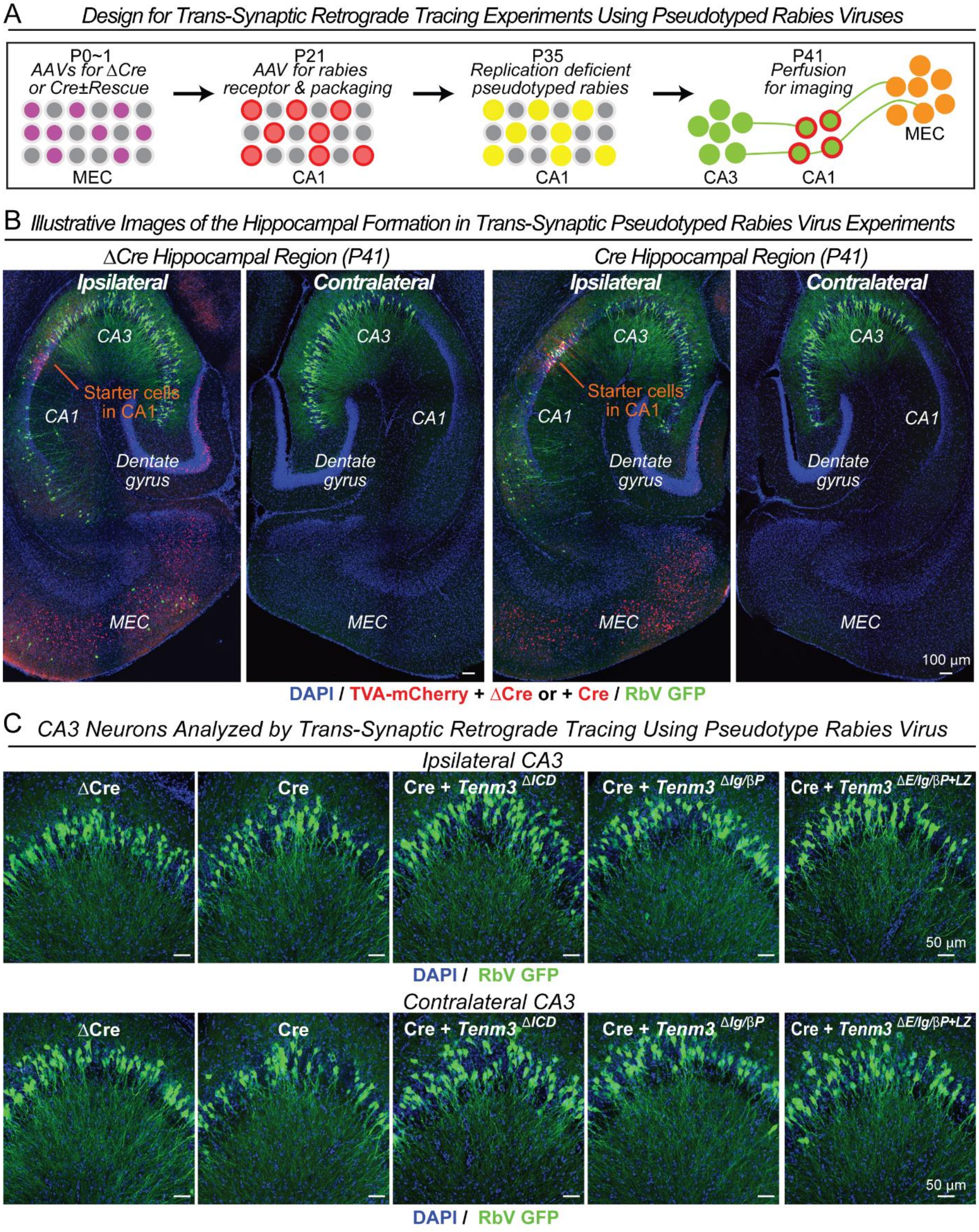

Fig. S11. Further characterization of retrograde trans-synaptic tracing experiments using pseudo-typed rabies viruses which were used to test the structure-function relation of Tenm3 in synapse formation *in vivo*

(**A**) Experimental strategy that enables testing of the effect of presynaptic Cre-mediated gene deletions on synaptic connections using retrograde pseudo-typed rabies virus tracing methods (*25, 31*). Medial entorhinal cortex (MEC) neurons of Tenm3/4 double cKO mice were infected unilaterally at P0 with AAVs expressing tdTomato-tagged ΔCre (control) or tdTomato-tagged Cre alone, or the Cre together with HA-tagged mutant Tenm3 constructs ($Tenm3^{\Delta ICD}$, $Tenm3^{\Delta Ig/\beta p}$, and $Tenm3^{\Delta E/Ig/\beta P+LZ}$). Proximal CA1 neurons were infected at P21 with AAVs encoding Cre-independent mCherry-tagged rabies virus receptor and rabies virus packaging proteins. The same proximal CA1 neurons were then infected at P35 with pseudo-typed rabies viruses encoding eGFP. Finally, the presynaptic inputs from the ipsi- and contralateral hippocampal CA3 region and the ipsilateral MEC were analyzed at P41 by imaging.

(**B**) Representative images of a pseudo-typed rabies virus retrograde tracing experiment analyzing the effect of a presynaptic Tenm3/4 deletion on synaptic inputs onto CA1 neurons (tdTomato (red) signal in the MEC from ΔCre and Cre; mCherry (red) signal in the CA1 from the pseudo-typed rabies virus receptor (starter cells); eGFP (green) signal visualizing presynaptic inputs onto CA1 neurons; merged images including a nuclear DAPI (blue) stain).

(**C**) Representative images of the ipsi- (top) and contralateral (bottom) hippocampal CA3 neurons analyzed by trans-synaptic retrograde tracing using pseudo-typed rabies virus.

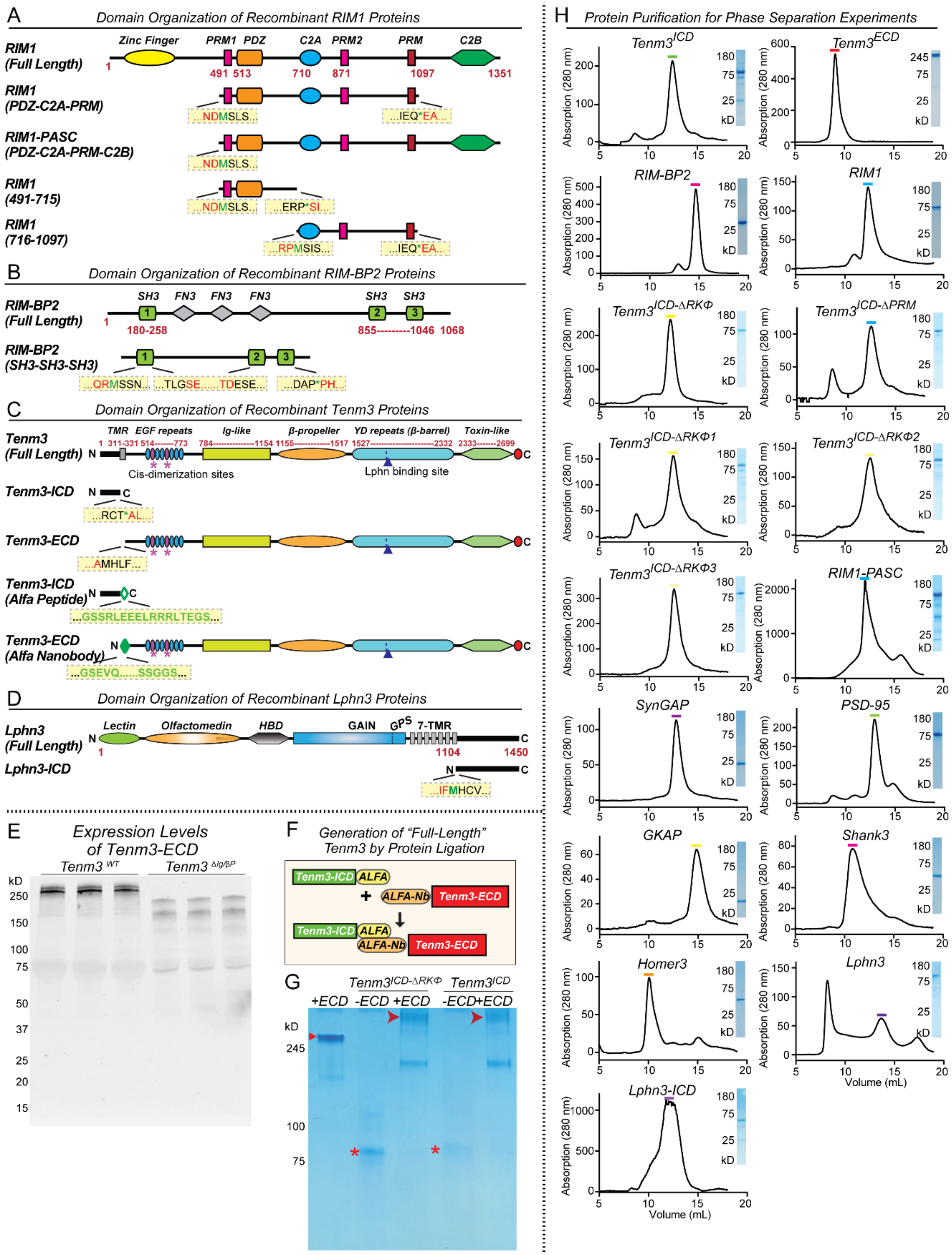

Fig. S12. Domain structures of proteins used in liquid-liquid phase-separation (LLPS) experiments (A to D), rationale and strategy for producing recombinant full-length Tenm3 protein (E to G), and characterization of purified proteins used for LLPS experiments (H)

(**A** to **D**) Domain organization of recombinant proteins used for phase-separation experiments (A, RIM1; B, RIM-BP2; C, Tenm3; D, Lphn3).

(**E**) SDS-polyacrylamide gel documenting that recombinant expression of the full-length extracellular regions of wild-type Tenm3 (*Tenm3^{WT}*) is more efficacious than that of the minimal Tenm3 construct containing a deletion of the Ig-like and β-propeller domains (*Tenm3$^{\Delta Ig/\beta P}$*) (see Fig. 1 and figs. S5 and S6).

(**F**) Schematic of the generation of recombinant 'full-length' Tenm3 protein lacking a TMR using nanobody-mediated ligation. Tenm3^{ECD} and Tenm3^{ICD} proteins are ligated by the alfa-tag/alfa nanobody interaction to generate full-length Tenm3. This was necessary because the expression levels of full-length Tenm3 containing a TMR in HEK293 cells (panel E) was insufficient for purification of recombinant protein.

(**G**) Coomassie stained native polyacrylamide gel illustrating the ligation of Tenm3^{ECD} and Tenm3^{ICD} (wide-type and ΔRKΦ) mediated by the alfa-tag/alfa nanobody interaction that exhibits a high enough affinity to render the binding effectively irreversible. The triangular arrow marks Tenm3^{ECD}, the asterisks Tenm3^{ICD}, and the arrowheads Tenm3$^{ICD\text{-}ECD}$.

(**H**) Size exclusion chromatographs and Coomassie-stained gels (insets) of proteins used for phase-separation experiments. Peak fractions were analyzed by SDS-PAGE and Coomassie staining (insets on the right) and used as final purified proteins for phase separation experiments.

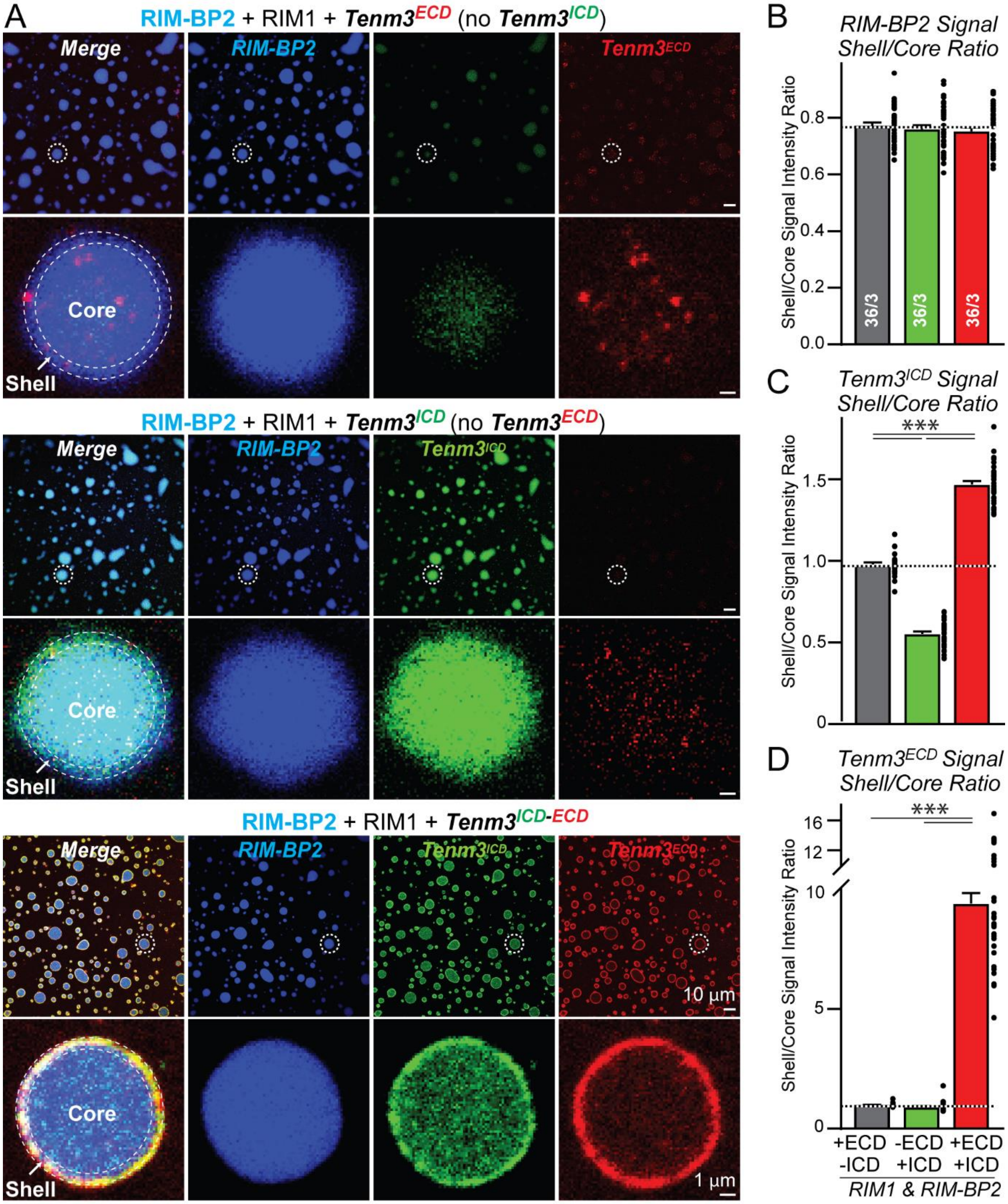

Fig. S13. Documentation of the selective recruitment of the Tenm3 ICD into phase-separated presynaptic active zone LLPS condensates, with a description of the quantification approach that was used to differentiate between the core and the shell of LLPS condensates

(**A**) Representative images complementing those shown in Fig. 2B illustrating how the shell (surface) and core (interior) LLPS condensate signals were quantified. Tenm3^{ECD} is not by itself recruited to active zone LLPS condensates (top set), whereas Tenm3^{ICD} is fully integrated into the

LLPS condensate core when added without the ECD (middle set), but is pulled to the surface shell when added in the context of the ECD in the Tenm3$^{ICD\text{-}ECD}$ protein (bottom set). For each set of images, top panels depict low magnification images of the LLPS condensates and bottom panels zoomed-in high-magnification images of the LLPS condensate that is circled in the low magnification images.

(**B** to **D**) Quantifications of the shell-to-core ratio for RIM-BP2 (B), the Tenm3 ICD (C) and the Tenm3 ECD (D) in Fig. 2B and in panel A. Data are means ± SEM (numbers of cells and experiments are indicated in bars in B), with ***P < 0.001, **P < 0.01, *P < 0.05 [one-way ANOVA with post-hoc Tukey tests].

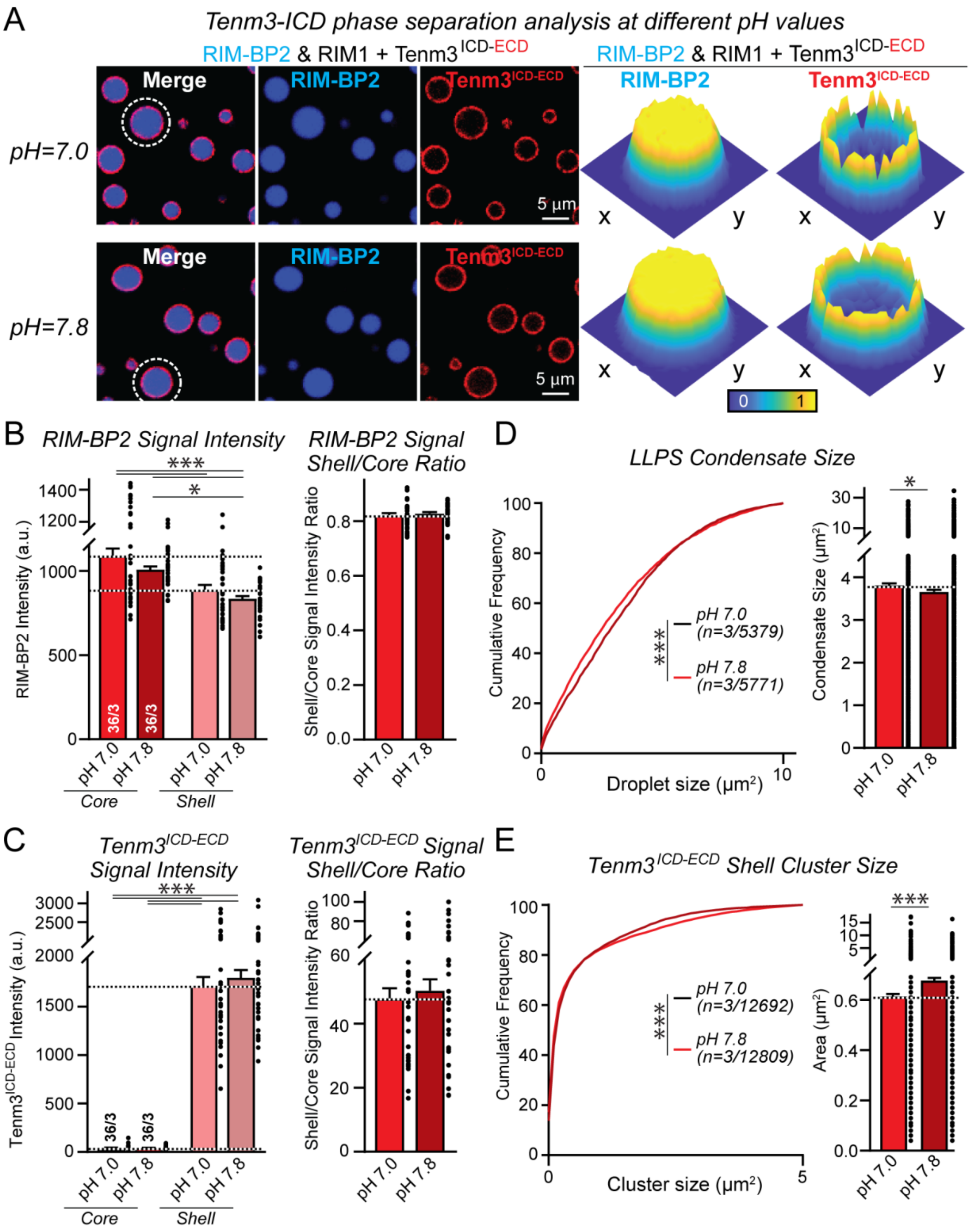

Fig. S14. The Tenm3 ICD incorporates into phase-separated presynaptic active zone LLPS condensates at both the standard pH (7.0) and at an elevated pH (7.8)

(**A**) Representative images (left) and heatmaps (right, corresponding to the circled condensates on the left) demonstrating the recruitment of the Tenm3^{ICD} to RIM1 and RIM-BP2 (both 10 µM)

LLPS condensates at different pH values. RIM-BP2 and Tenm3^{ECD} were labeled by iFluor-488 and iFluor-546, respectively, while RIM1 was unlabeled.

(**B** and **C**) Quantifications of RIM-BP2 (B) and Tenm3^{ECD} (C) signals across phase-separated RIM1 and RIM-BP2 LLPS condensates illustrating that pH values have no effect on the shell-to-core ratio of RIM-BP2 and Tenm3^{ECD} (left, absolute signal intensity; right, ratio of shell to core signal with 'control' signals constituting background).

(**D**) Cumulative probability plot (left) and summary graph (right) of the active zone LLPS condensate sizes as a function of two pH values.

(**E**) Cumulative probability plot (left) and summary graph (right) of the sizes of 'puncta' (referred to as 'shell cluster') formed by the Tenm3$^{ICD\text{-}ECD}$ on the surface shell of presynaptic active zone LLPS condensates as a function of pH.

Numerical data are means ± SEM (numbers of condensates and experiments are indicated in bars). ***$P < 0.001$, **$P < 0.01$, *$P < 0.05$ [B and C left: Two-way ANOVA with post-hoc Tukey tests; D, E left: Kolmogorov–Smirnov t test; B, C, D, and E right: two-tailed t test]. a.u., arbitrary units.

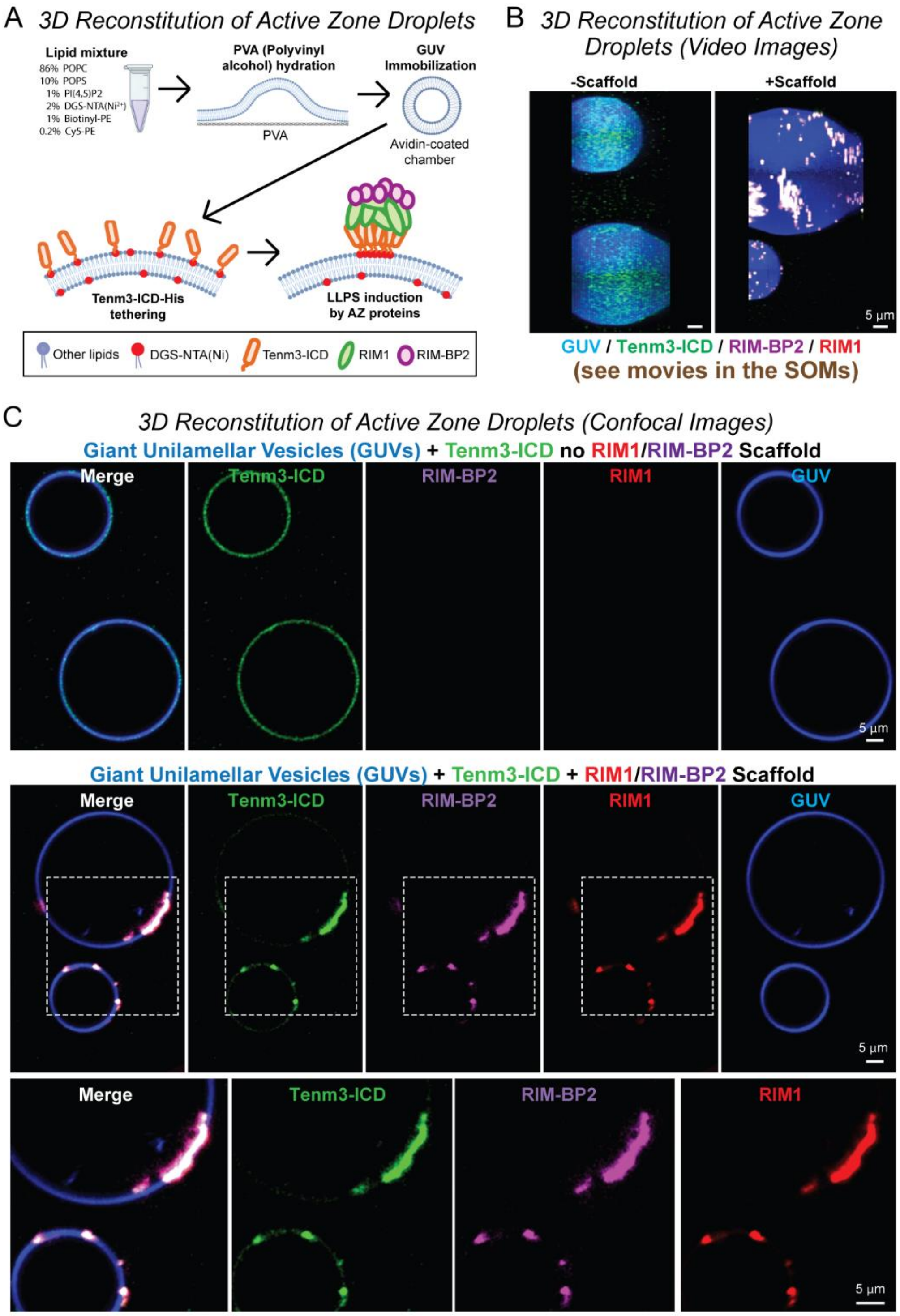

Fig. S15. Recruitment of active zone LLPS condensates to the surface of giant unilamellar vesicles (GUVs) by the Tenm3 ICD that is tethered to the lipid bilayer

(**A**) Experimental strategy. Fluorescently labeled purified Tenm3 ICD protein containing a hexa-His tag is anchored to the lipid bilayer of GUVs by binding to modified lipids with a Ni^{2+}-containing headgroup. Fluorescently labeled active zone LLPS condensates are subsequently

added to the GUVs containing the Tenm3 ICD and the recruitment of the LLPS condensates is imaged by confocal microscopy.

(**B**) Three-dimensional reconstruction using confocal microscopy of a GUV containing the tethered Tenm3 ICD alone (left) or the Tenm3 ICD with recruitment of active zone LLPS condensates (2 µM RIM1 and RIM-BP2) (right). See Supplementary Movies #1 and #2 for a better view.

(**C**) Two-dimensional images of GUVs containing the tethered Tenm3 ICD in the absence (top) or presence of RIM1/RIM-BP2 (middle), with a higher magnification of the phase-separated active zone LLPS condensates shown at the bottom. In the absence of active zone LLPS condensates, the Tenm3 ICD is evenly distributed over the GUV surface, whereas in the presence of active zone LLPS condensates, the Tenm3 ICD becomes clustered in small patches (‘puncta’) of membrane containing the active zone LLPS condensates.

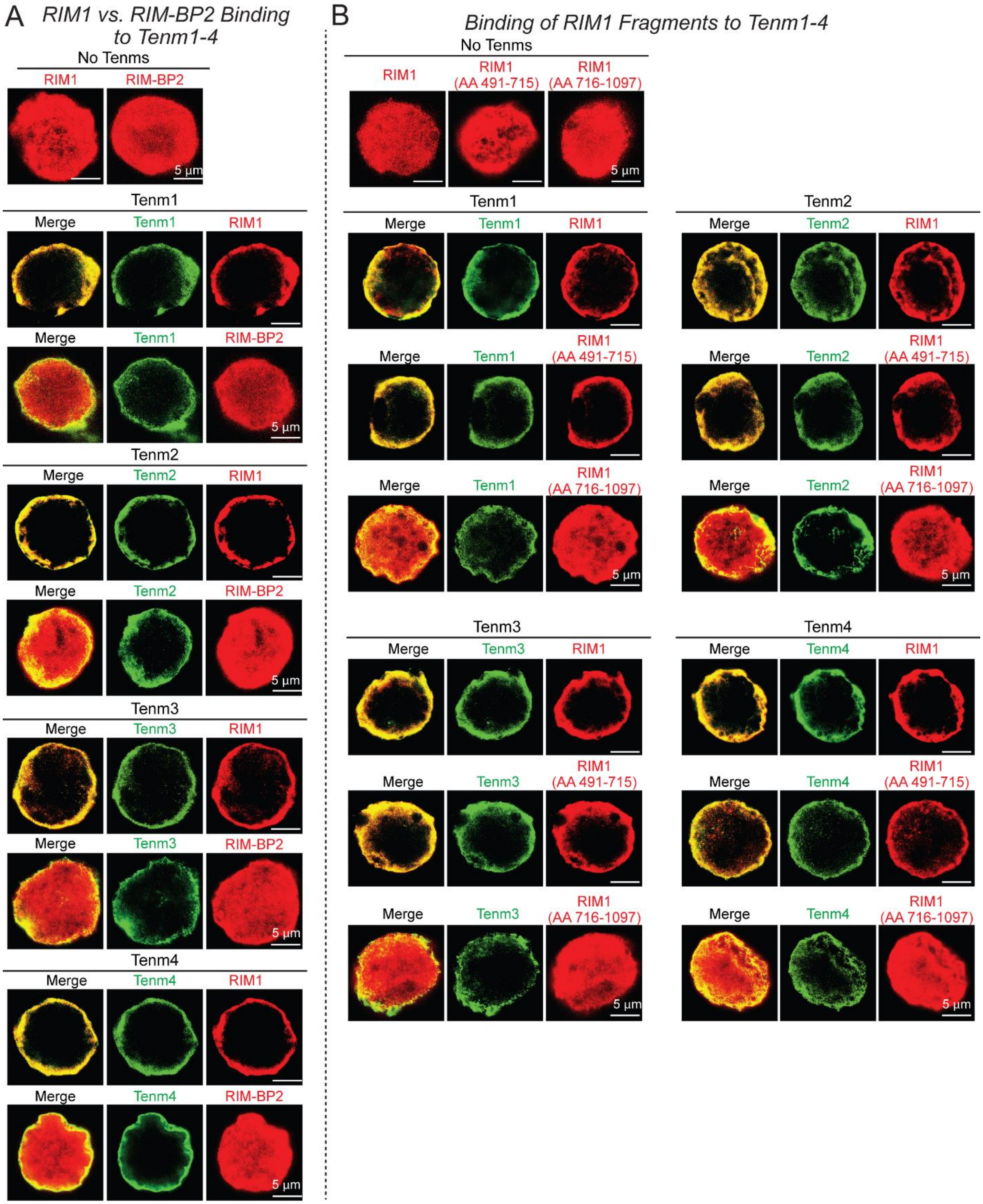

Fig. S16. Plasma membrane recruitment assays in transfected HEK293T cells reveal domain-specific interactions between Temm1-4 and RIM1 but not RIM-BP2

(**A**) Imaging of transfected HEK293T cells co-expressing Flag-tagged RIM1 or RIM-BP2 with HA-tagged Tenm1-4 (red, Flag-epitope labeling; green, HA-epitope labeling; for domain

structures, see fig. S11), illustrating that all teneurin isoforms recruit RIM1 but not RIM-BP2 to the plasma membrane, whereas RIM1 alone does not localize to the plasma membrane.

(**B**) Imaging of transfected HEK293T cells co-expressing different fragments of Flag-tagged RIM1 (amino acids 491-715 or 716-1097) with HA-tagged Tenm1-4 (red, Flag-epitope; green, HA-epitope), illustrating that all teneurin isoforms bind to the RIM1 fragment containing amino acids 491-715 but not to the RIM1 fragment containing amino acids 716-1097.

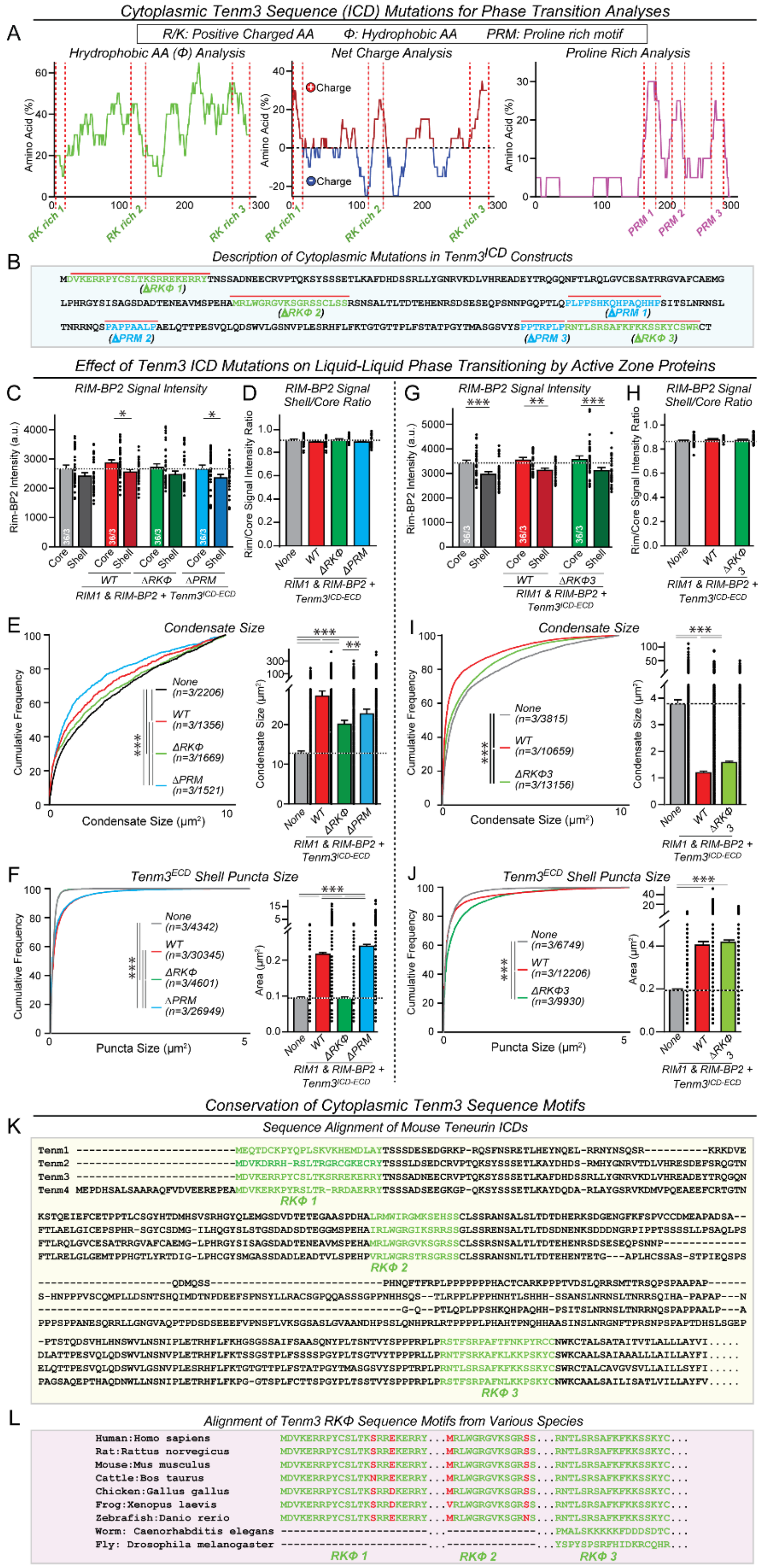

Fig. S17. Analyses of Tenm3 ICD sequences to identify potential LLPS-forming sequence motifs (A), description of the mutations in the Tenm3 ICD that were examined in LLPS

formation experiments (B), further characterization of the effect of these mutations on the recruitment of the Tenm3 ICD into active zone LLPS condensates (C to J), and sequence alignments demonstrating the conservation of critical LLPS motifs in all teneurins (K and L)

(**A**) Analysis of the Tenm3^{ICD} amino acid composition for identification of LLPS-forming sequence motifs (R/K, arginine/lysine rich; Φ, hydrophobic residues; PRM, proline rich motif).

(**B**) Description of the Tenm3^{ICD} deletion mutations that were examined in phase separation experiments. The sequences identified by red lines were deleted in the various mutations, with the 'ΔRKΦ' and the 'ΔPRM' mutants of the Tenm3 ICD including all three mutations.

(**C** to **E**) Summary graphs of the overall signal intensity (C) and the shell-to-core ratio (D) of the RIM-BP2 signal and cumulative probability plot of the active zone LLPS condensate size (E) as a function of the addition of wild-type or mutant Tenm3^{ICD}, extending the data shown in Fig. 4B. Various Tenm3^{ICD} proteins have no discernible effect on the structure of presynaptic active zone protein condensates but induce small, significant shifts in condensate sizes.

(**F**) Quantification of the size of 'puncta' formed by the Tenm3^{ECD} on the surface shell of the presynaptic active zone protein condensates as a function of the addition of wild-type and mutant Tenm3 ICDs.

(**G** to **J**) Same as C to F but analyzing the effect of the ΔRKΦ3 mutation on presynaptic active zone protein condensates.

(**K**) Sequence alignment of the cytoplasmic ICDs of the four mouse teneurins (Tenm1-4) to demonstrate the conservation of the arginine/lysine rich motifs (RKΦ) in teneurins.

(**L**) Sequence alignment of the three arginine/lysine rich motifs in Tenm3 sequences from different species.

Numerical data in C to J are means ± SEM (numbers of condensates and experiments are indicated in bars). ***$P < 0.001$, **$P < 0.01$, *$P < 0.05$ [C and G: two-way ANOVA with post-hoc Tukey tests; D, E right, F right, H, I right, and J right: one-way ANOVA with post-hoc Tukey tests; E left, F left, I left, and J left: Kolmogorov–Smirnov t test].

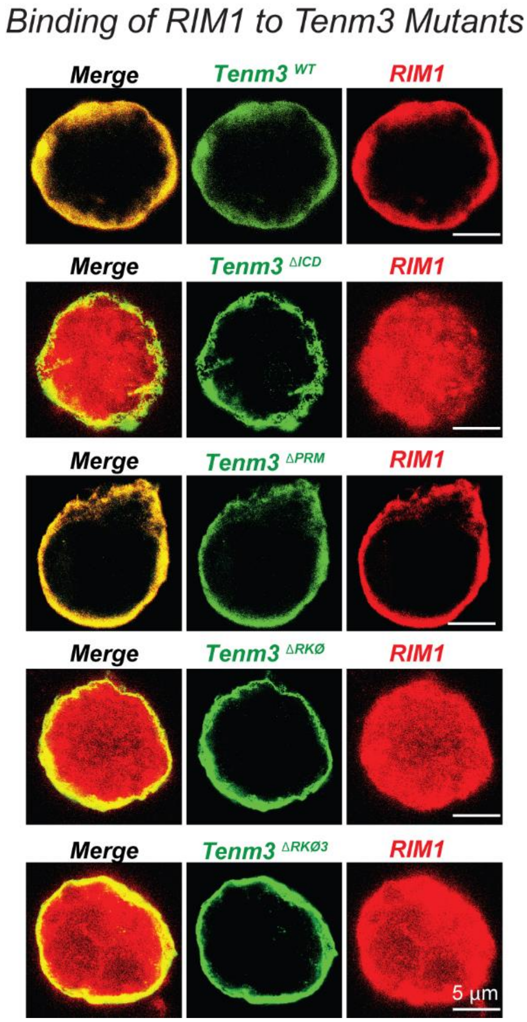

Fig. S18. The Tenm3^{ICD} ΔRKΦ mutations impair binding of the Tenm3^{ICD} to RIM1. Imaging of transfected HEK293T cells co-expressing wild-type and mutant HA-tagged Tenm3 with Flag-tagged RIM1 (red, Flag-epitope; green, HA-epitope) without co-expression of RIM-BP2. The ΔRKΦ and ΔRKΦ3 mutations severely impair recruitment of RIM1 to the cell surface.

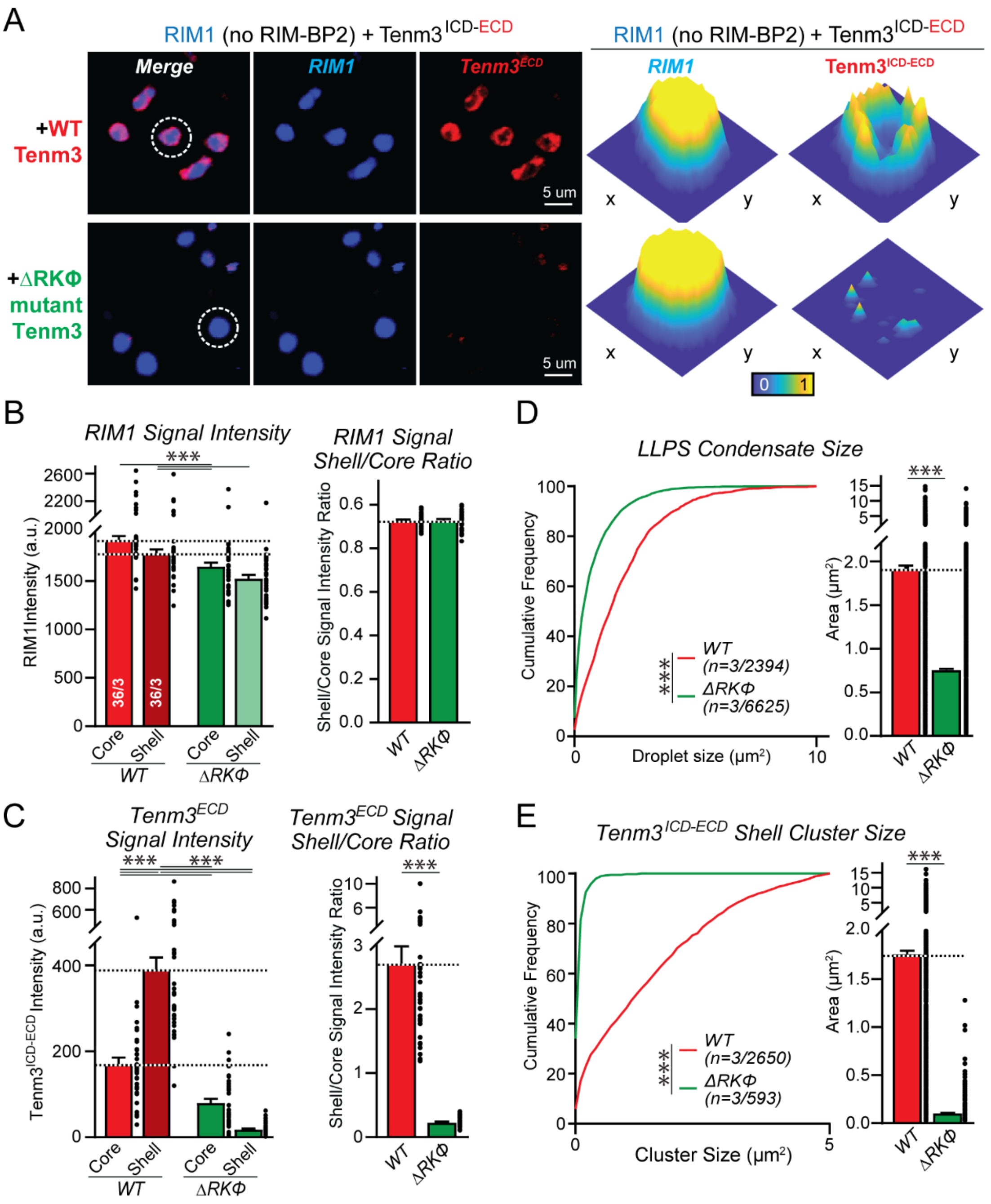

Fig. S19. Sequence-specific recruitment of Tenm3$^{ICD-ECD}$ to LLPS condensates formed only by RIM1 to further document the validity of the Tenm3^{ICD} interaction with RIM1

(**A**) Representative images (left) and heatmaps (right, corresponding to the circled condensates on the left). As shown previously (*8*), RIM1 (10 μM) can form LLPS condensates in the absence of RIM-BP2 that are smaller in size. The RIM1-only LLPS condensates also effectively recruit wild-type but not ΔRKΦ-mutant Tenm3$^{ICD-ECD}$ to the shell of the LLPS condensates. RIM1 and Tenm3^{ECD} were labeled with iFluor-488 and iFluor-546, respectively. Tenm3^{ICD} was unlabeled and is thus invisible. A longer RIM1 fragment was used here for efficient LLPS formation (the

'PASC' fragment; fig. S11) than the fragment used in experiments with active zone LLPS condensates formed by both RIM1 and RIM-BP2.

(**B** and **C**) Quantification of RIM1 (B) and $Tenm3^{ICD\text{-}ECD}$ signals (C) across phase-separated RIM1 LLPS condensates illustrating that $Tenm3^{ICD\text{-}ECD}$ is recruited into the condensates while the ΔRKΦ mutations abolish such recruitment (left in B and C, absolute signal intensity; right, ratio of shell to core signal with 'control' signals constituting background).

(**D**) Cumulative frequency plot and summary graph of the active zone protein condensate size as a function of the RKΦ3 mutation.

(**E**) Quantification of the size of 'puncta' formed by the $Tenm3^{ICD\text{-}ECD}$ on the surface shell of the presynaptic active zone protein condensates as a function of the RKΦ3 mutation.

Numerical data are means ± SEM (numbers of condensates and experiments are indicated in bars). ***$P < 0.001$, **$P < 0.01$, *$P < 0.05$ [B and C left: Two-way ANOVA with post-hoc Tukey tests; D, E left: Kolmogorov–Smirnov t test; B, C, D, and E right: two-tailed t test]. a.u., arbitrary units.

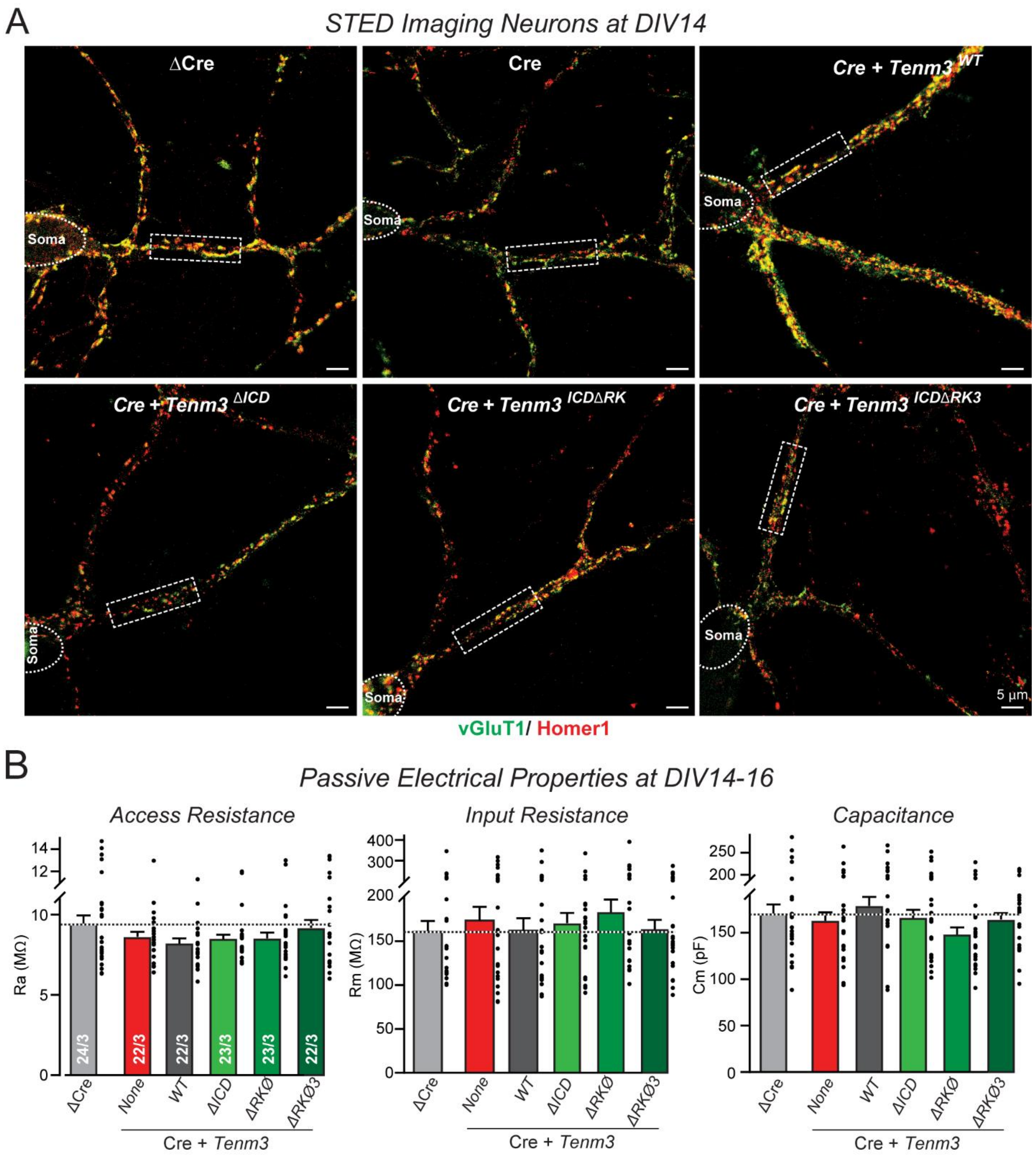

Fig. S20. Additional data for Fig. 4 on the functional analysis of Tenm3^{ICD} mutations

(**A**) Representative low-magnification STED super-resolution images corresponding to Fig 4A.

(**B**) Summary graphs of the passive electrical properties of the neurons analyzed in Fig. 4E and F demonstrating that the manipulation of Tenm3 in the Tenm3/4 double deletion cortical neurons has no effect on passive electrical properties (access resistance, capacitance, input resistance) of neurons at DIV14-16. Data are means ± SEM (numbers of cells and experiments are indicated in bars), with no detected statistical significance using one-way ANOVA with post-hoc Dunnett tests.

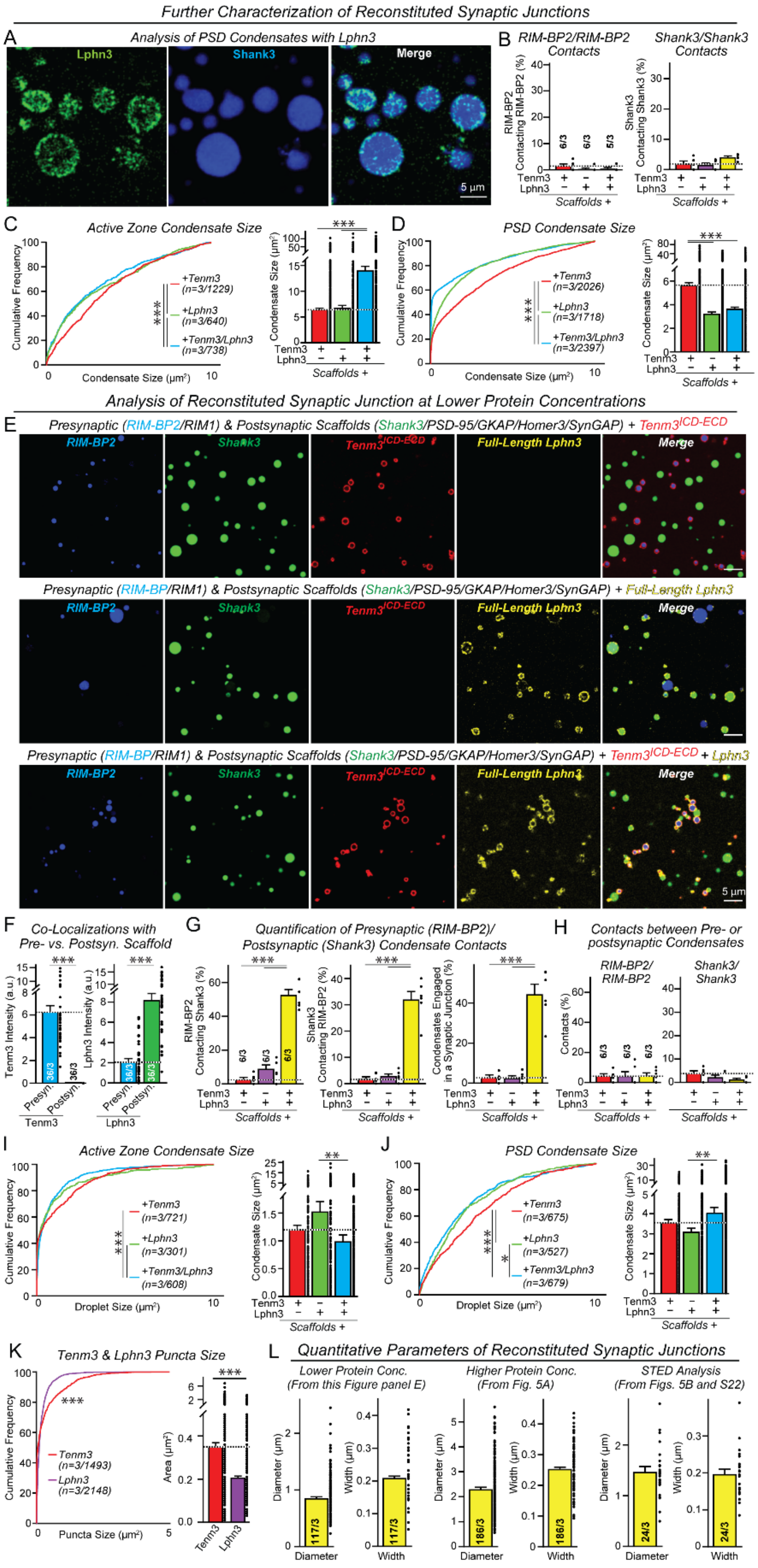

Fig. S21. Further characterization of reconstituted synaptic junctions (A to D), independent replication of the reconstitution of synaptic junctions at a lower protein concentration (E to K), and quantifications of the size of reconstituted synaptic junctions (L)

(**A**) Representative images illustrating that Lphn3 (green) is enriched on the surface of postsynaptic scaffold condensates visualized using labeled Shank3 (blue) as described (*32*).

(**B**) Quantification of the contacts between presynaptic active zone LLPS condensates (RIM-BP2) or between postsynaptic PSD scaffold LLPS condensates (Shank3) when incubated either alone or together to document that these LLPS condensates cannot form contacts with themselves (corresponding to Fig 5A).

(**C** and **D**) Quantification of the sizes of phase-transitioned LLPS condensates formed by presynaptic active zone proteins RIM1/RIM-BP2 (C) or postsynaptic scaffold proteins Shank3/PSD-95/GKAP/Homer3/SynGAP (D) as a function of the addition of Lphn3 and Tenm3$^{ICD\text{-}ECD}$ (corresponding to Fig 5A).

(**E**) Representative images of active zone and postsynaptic density protein LLPS condensates formed at lower protein concentrations in the presence of purified either Tenm3$^{ICD\text{-}ECD}$ (top row), full-length Lphn3 (middle row), or both (bottom row) to document that LLPS condensates can form and recruit Tenm3 and Lphn3 independent of the protein concentration. Proteins and concentrations used: truncated RIM1 and RIM-BP2, both 10 μM; full-length PSD-95, truncated GKAP with an DLS sequence at the GK binding region as a phospho-mimicking mutation to enhance binding to PSD-95, truncated SynGAP, truncated Shank3, and full-length Homer3, all 2.5 μM; Tenm3^{ICD}, Tenm3^{ECD} and full-length Lphn3 were at 0.5 μM.

(**F**) Quantification of the co-localization of Tenm3$^{ICD\text{-}ECD}$ and Lphn3 with pre- vs. postsynaptic LLPS condensates to determine the degree of specificity of the recruitment of Tenm3$^{ICD\text{-}ECD}$ proteins to presynaptic active zone LLPS condensates at the lower protein concentration of panel E (left, labeled via RIM-BP2) and of Lphn3 to the postsynaptic LLPS condensates (right, labeled via Shank3).

(**G**) Quantification of contacts between presynaptic active zone (labeled via RIM-BP2) and PSD scaffold LLPS condensates (labeled via Shank3) as a function of the addition of either Tenm3$^{ICD\text{-}ECD}$ or full-length Lphn3 or both to test whether at a lower protein concentration (same as panel E) either Tenm3 or Lphn3 are sufficient to connect the separate active zone and postsynaptic LLPS condensates into junctions.

(**H**) Same a panel B, but for the lower protein concentration under the conditions described for panel E.

(**I** and **J**) Same a panels C and D, but for the lower protein concentration under the conditions described for panel E.

(**K**) Quantification of the sizes of the 'puncta' formed by Tenm3$^{ICD\text{-}ECD}$ and Lphn3 on the surface shell of active zone and postsynaptic density LLPS condensates, respectively, when they are recruited to these condensates. Data are from the experiments in panel E under conditions of adding either Tenm3$^{ICD\text{-}ECD}$ or Lphn3 alone to the condensates.

(**L**) Quantifications of the diameter and apparent width of reconstituted synaptic junctions at different protein concentrations. The parameters were quantified in profiles of reconstituted synaptic junctions using STED images with high x- and y- but lower z-dimension resolutions (see fig. S23 for additional examples). The diameter was measured as the length of the junction delineated by the Tenm3-Lphn3 complexes, whereas the width was measured as the distance between the peak Tenm3 and Lphn3 signals at a junction. Since the junctions are not completely planar but curved and are imaged at an imperfect perpendicular angle, the width measurements

represent the sum of z-signals and thus do not reflect the true average junction width. The width measurements are only displayed to estimate reproducibility of junction properties between different experiments. The diameter measurements, conversely, are likely accurate since they are independent of the z-dimension, imaging angle and junction curvature.

Numerical data are means ± SEM (numbers of droplets and experiments are indicated in bars). ***$P < 0.001$, **$P < 0.01$, *$P < 0.05$ [B, C right, D right, G, H, I right, and J right: One-way ANOVA with post-hoc Tukey tests; C left, D left, I left, J left, and K left: Kolmogorov–Smirnov t test; F and K right: two-tailed t test].

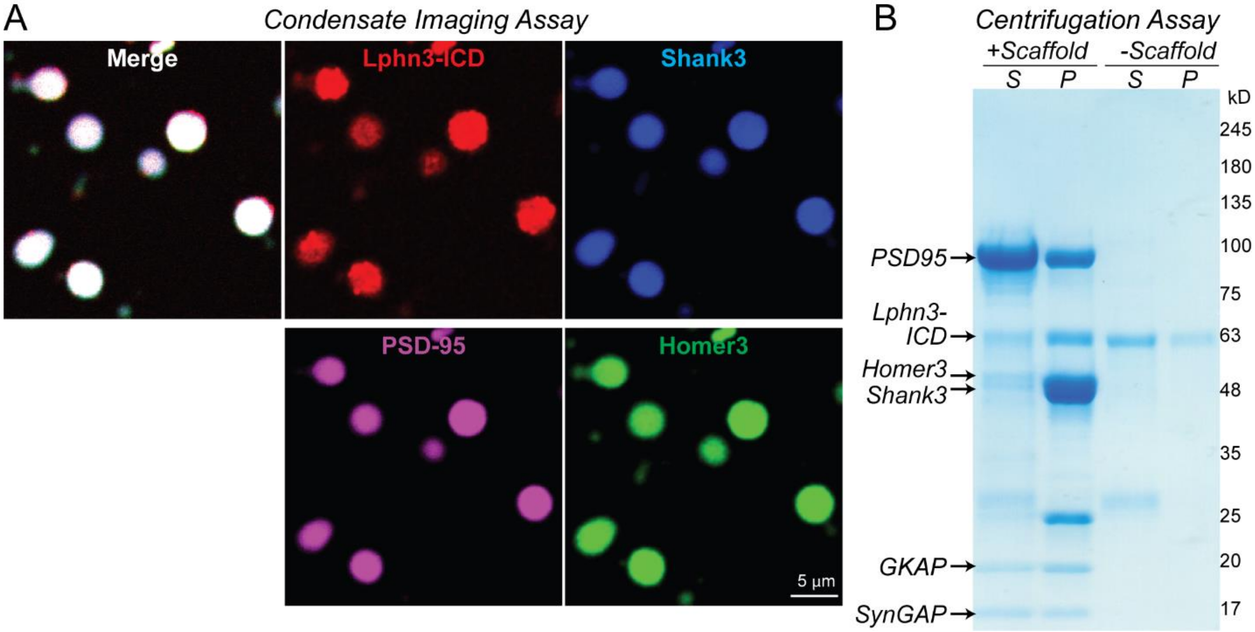

Fig. S22. The Lphn3 intracellular domain (ICD) (exon 31 splice variant [*32*] quantitatively inserts into liquid-liquid phase-separated (LLPS) condensates formed by postsynaptic density scaffold proteins

(**A**) Representative images illustrating the recruitment of Lphn3^{ICD} (0.5 µM) to PSD condensates with PSD-95, Homer3, truncated GKAP with a DLS sequence at the GK binding region (a phospho-mimicking mutation to enhance PSD-95 binding), SynGAP, and Shank3 (all 5 µM). Lphn3^{ICD}, Shank3, PSD-95, and Homer3 were labeled by iFluor-546, iFluor-405, Alexa 647, and iFluor-488, respectively.

(**B**) Sedimentation assay of liquid-liquid phase transitioned PSD condensates provides independent confirmation that Lphn3^{ICD} can be recircuited into condensates.

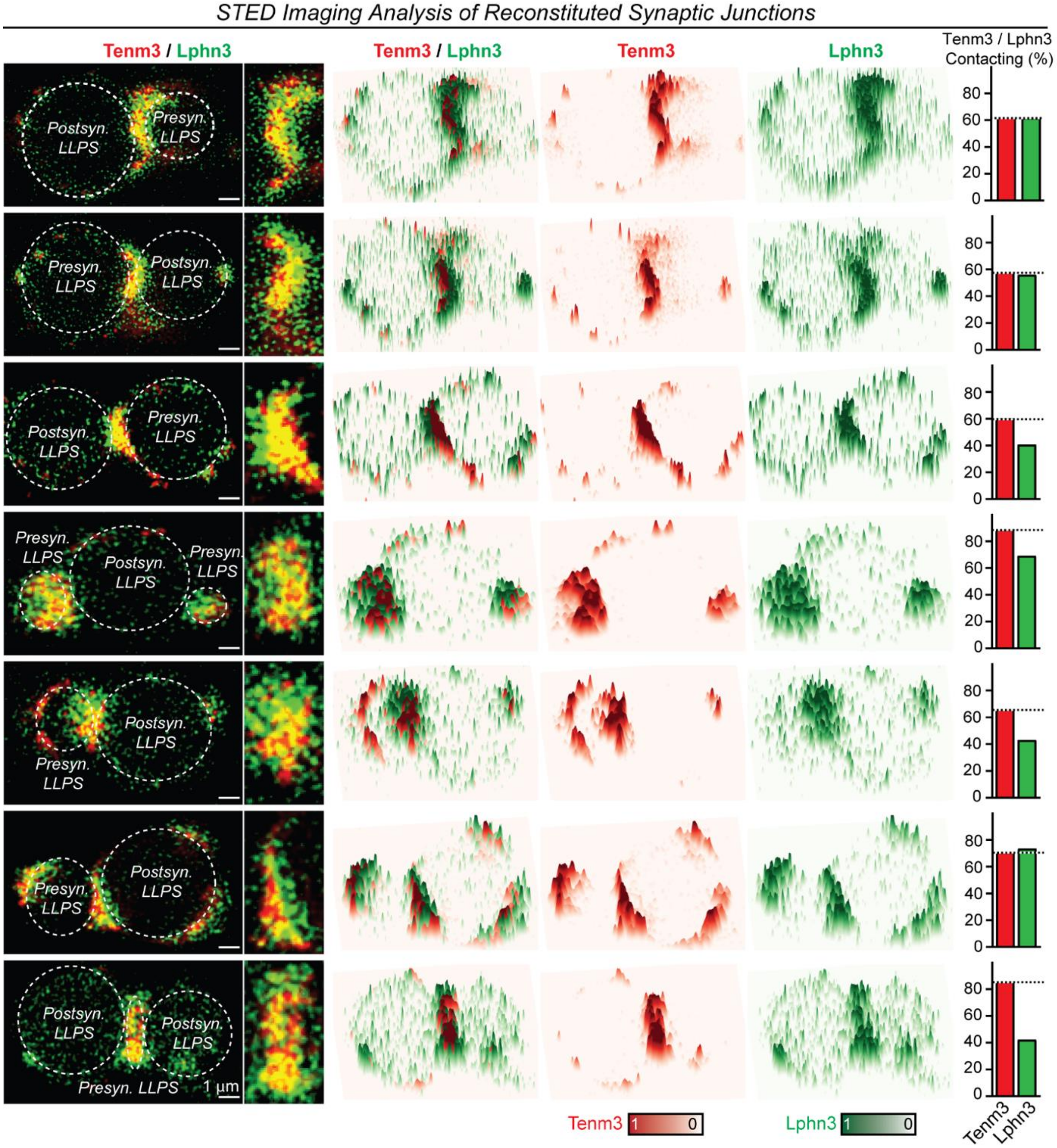

Fig. S23. Representative images of individual reconstituted synaptic junctions

The images show further analyses complementing the analyses of Fig. 5B. For all rows: Left, high-magnification STED super-resolution images; middle, 3D rendering heatmaps of the distribution of densely clustered Tenm3$^{ICD-ECD}$ and Lphn3 complexes at the contact sites of phase-separated presynaptic active zone and PSD condensates to visualize the enrichment of Tenm3$^{ICD-ECD}$ and Lphn3 at condensate junctions; right, summary graphs of the percentage of Tenm3 contacts with Lphn3 and vice versa to depict experimental variations. As in Fig. 5B, the fluorescence signal imaged here is derived from fluorescently labeled, purified recombinant Lphn3 and Tenm3 proteins that are combined with presynaptic active zone LLPS condensates and postsynaptic scaffold protein LLPS condensates, which don't mix.

SUPPLEMENTARY MOVIES

Movie S1

Three-dimensional reconstruction of a giant unilamellar vesicle coated with the Tenm3 ICD

Three-dimensional reconstruction using confocal microscopy of a GUV containing the tethered Tenm3 ICD in the absence of RIM1/RIM-BP2 LLPS condensates. The Tenm3 ICD is evenly distributed over the GUV surface (Blue, GUV; Green, Tenm3-ICD).

Movie S2

Three-dimensional reconstruction of a giant unilamellar vesicle coated with the Tenm3 ICD that has recruited active zone LLPS condensates

The same reconstruction as in Movie #1 except that in the presence of RIM1/RIM-BP2 (2 µM) on the GUV surface. The Tenm3 ICD becomes recruited to small patches of membrane containing the RIM1/RIM-BP2 LLPS condensates (Blue, GUV; Green, Tenm3-ICD; Magenta, RIM-BP2; Red, RIM1).
